# Establishment of Trophectoderm Cell Lines from Buffalo (*Bubalus bubalis*) Embryos of Different Sources and Examination of In Vitro Developmental Competence, Quality, Epigenetic Status and Gene Expression in Cloned Embryos Derived from Them

**DOI:** 10.1371/journal.pone.0129235

**Published:** 2015-06-08

**Authors:** Sushil Kumar Mohapatra, Anjit Sandhu, Karn Pratap Singh, Suresh Kumar Singla, Manmohan Singh Chauhan, Radheysham Manik, Prabhat Palta

**Affiliations:** Animal Biotechnology Centre, National Dairy Research Institute, Karnal, India; The Babraham Institute, UNITED KINGDOM

## Abstract

Despite being successfully used to produce live offspring in many species, somatic cell nuclear transfer (NT) has had a limited applicability due to very low (>1%) live birth rate because of a high incidence of pregnancy failure, which is mainly due to placental dysfunction. Since this may be due to abnormalities in the trophectoderm (TE) cell lineage, TE cells can be a model to understand the placental growth disorders seen after NT. We isolated and characterized buffalo TE cells from blastocysts produced by in vitro fertilization (TE-IVF) and Hand-made cloning (TE-HMC), and compared their growth characteristics and gene expression, and developed a feeder-free culture system for their long-term culture. The TE-IVF cells were then used as donor cells to produce HMC embryos following which their developmental competence, quality, epigenetic status and gene expression were compared with those of HMC embryos produced using fetal or adult fibroblasts as donor cells. We found that although TE-HMC and TE-IVF cells have a similar capability to grow in culture, significant differences exist in gene expression levels between them and between IVF and HMC embryos from which they are derived, which may have a role in the placental abnormalities associated with NT pregnancies. Although TE cells can be used as donor cells for producing HMC blastocysts, their developmental competence and quality is lower than that of blastocysts produced from fetal or adult fibroblasts. The epigenetic status and expression level of many important genes is different in HMC blastocysts produced using TE cells or fetal or adult fibroblasts or those produced by IVF.

## Introduction

The mammalian blastocyst is composed of two types of cell populations, the inner cell mass (ICM), which gives rise to the embryo and its associated membranes, and the trophectoderm (TE), which forms the extra-embryonic tissues of the placenta. TE cells are the first to differentiate, and their differentiation is necessary for pregnancy recognition, implantation and formation of placenta [1]. However, the processes that regulate these developmental milestones are not well understood due to involvement of a multitude of participating factors and complex interactions among them. The placenta of bovidae animals contain two types of cells, the mono- or uninucleate and the binucleate cells, the former are responsible for the production of interferon-tau (IFN-τ), which is also produced by the TE of peri-implantation blastocysts [2]. Production and secretion of IFN-τ is necessary for the successful pregnancy since high levels of IFN-τ expression attained at the time of implantation act as a pregnancy recognition signal [3]. Despite being successfully used to produce live offspring in many farm animal species, somatic cell nuclear transfer (NT) has had a limited applicability due to very low overall cloning efficiency. Less than 1% of reconstructed embryos have been reported to give rise to live offspring across all species [4]. This is primarily because of a high incidence of pregnancy failure and accompanying placental and fetal pathologies. Pre- and early post-implantation losses can affect up to 70% of the pregnancies whereas in the surviving pregnancies, placentomegaly and fetal overgrowth are commonly observed [5]. It has been suggested that some fetal abnormalities observed in cloned calves, such as enlarged heart, enlarged umbilical cord, and abdominal ascites are consequences of placental dysfunction and, therefore, the condition described by the term "large offspring syndrome" has been suggested to be better described by "large placenta syndrome," because this syndrome affects an average of 50% of late-gestation NT pregnancies [6]. A similar pattern has been reported in sheep also [7]. The placenta is believed to be central to the onset of the pathologies associated with pregnancies from NT embryos. Since the placental abnormalities may be primarily due to those in the TE cell lineage, TE cells can be a model to understand the placental growth disorders that are seen after NT.

Isolation of TE or trophoblast cells from placenta or choriocarcinoma or in vitro fertilized (IVF) embryos and their culture has been reported in several species such as cattle [8,9], goat [10], pig [11], rat [12] and human [13]. Trophoblast cell lines derived from cattle embryos produced in vivo or by IVF, NT or parthenogenesis have been compared for their characteristics in many studies [14,15,16,17,18,19]. It has been shown in several studies that IFN-τ production from primary trophectoderm outgrowths or cultures of parthenogenesis- or NT-derived bovine embryos is significantly lower than that from outgrowths or cultures of embryos produced in vivo or those produced by IVF [14,18]. Also, the time of detection of IFN-τ mRNA is delayed in NT embryos since its expression could be firstly detected at 16-cell stage in bovine IVF embryos on day 4, at morula stage in NT embryos on day 5 and at early blastocyst stage in parthenogenetically produced embryos on day 6, respectively, suggesting that IFN-τ mRNA expression patterns of bovine embryos derived from different procedures were different at the same development stage [20]. This raises the question whether the lower capability of NT embryos to produce IFN-τ is an indicator of their compromised potential to establish pregnancy following transfer to recipients. Since failure to form a normal placenta, which is believed to be a major cause of NT pregnancy loss and low IFN-τ production are both associated with TE cells, it should be explored whether the use of TE cells as donor cells could overcome these problems associated with NT embryos. However, after the first report in which the capability of TE cells to undergo reprogramming was demonstrated by the birth of live mouse pups [21], to our knowledge, there has been only one report on the use of TE cells as donor cells for NT [22].

We have standardized simple and efficient Hand-made cloning (HMC) technique in buffalo leading to the birth of offspring produced using different types of donor cells such as ear skin-derived somatic cells [23], embryonic stem cells [24] and semen-derived somatic cells [25]. Also, cloned buffalo embryos have been successfully produced using milk-derived cells [26]. However, in all these studies, the cloning efficiency achieved was very low. In the present study, we isolated and characterized buffalo TE cells from IVF hatched blastocysts (TE-IVF) and HMC blastocysts (TE-HMC), and compared their growth characteristics and gene expression, and developed a feeder-free system for their long-term in vitro culture. The TE-IVF cells were then used as donor cells to produce HMC embryos. The developmental competence, quality, epigenetic status and gene expression of these embryos were compared with those of HMC embryos produced using fetal fibroblasts (FF) or adult fibroblasts (AF) as donor cells.

## Materials and Methods

In vitro maturation (IVM) and IVF of oocytes and in vitro culture (IVC) of embryos and somatic cells was carried out at 38.5°C in a CO_2_ incubator (5% CO_2_ in air, 90–95% relative humidity). Animal experiments were carried out after approval by Committee for the Purpose of Control and Supervision on Experiments on Animals (Indian Council of Medical Research, New Delhi) and the Animal Ethics Committee (National Dairy Research Institute, Karnal).

### Production of embryos by IVF

Usable quality cumulus-oocyte complexes (COCs) obtained from slaughterhouse buffalo ovaries were subjected to IVM and IVF, as described earlier [27] with some modifications. Briefly, the COCs were washed several times with the IVM medium (TCM-199 + 10% FBS + 5 μg/mL pFSH + 1μg/mL estradiol-17β + 0.81 mM sodium pyruvate + 10% buffalo follicular fluid + 50μg/mL gentamicin sulphate) following which groups of 15–20 COCs were placed in 100 μL droplets of the IVM medium, overlaid with sterile mineral oil in 35 mm Petri dishes, and were cultured in a CO_2_ incubator for 24 h. For IVF, two straws of frozen-thawed buffalo semen, which had been tested for IVF earlier, were washed twice with the washing Bracket and Oliphant (BO) medium, containing 10 μg/mL heparin, 137.0 μg/mL sodium pyruvate and 1.942 mg/mL caffeine sodium benzoate. The pellet was re-suspended in 0.5 mL of the capacitation and fertilization BO medium (washing BO medium containing 10 mg/mL fatty acid-free BSA). The in vitro matured oocytes were washed twice with the fertilization BO medium and were then transferred to 50 μLdroplets (15–20 oocytes/droplet) of the capacitation and fertilization BO medium. The spermatozoa in 50 μL of the capacitation and fertilization BO medium (2–4 million spermatozoa/mL) were then added to the droplets containing the oocytes, covered with sterile mineral oil and were placed in a CO_2_ incubator for 18 h for IVF. After this, the cumulus cells were removed from the presumed zygotes by gentle pipetting. The presumed zygotes were then washed several times with Research Vitro Cleave medium (K-RVCL-50, Cook, Queensland, Australia) supplemented with 1% fatty acid-free BSA, and were cultured in this medium for up to 8 days post insemination in a CO_2_ incubator.

### Hand-made cloning (HMC)

For production of HMC embryos for isolation of TE cells, primary cell culture of skin cells derived from the ear biopsy of an adult Murrah buffalo was established and donor cell preparation was performed for HMC as reported earlier [28]. HMC was performed as described previously with some modifications [29]. Briefly, in vitro matured COCs were denuded by treatment with hyaluronidase (0.5mg/mL in T2 medium, where T denotes HEPES modified M-199 supplemented with 2.0 mM L-glutamine, 0.2 mM sodium pyruvate, 50 μg/mL gentamicin and the following number denotes 2% FBS) to remove cumulus cells. This was followed by zona digestion with treatment with pronase (2.0 mg/mL in T20 medium) for 10 min. The oocytes were then transferred to T20 medium and were kept at 38.5°C in a CO_2_ incubator for 10–15 min for visualization of the protrusion cone. Protrusion cone-guided bisection was performed in cytochalasin-B (2.5μg/mL in T20) using a microblade (MicroBlades, MTB-05; Micromanipulator Microscope Company, Inc. Washington DC, USA). The larger demicytoplasts without the protrusion cone were selected for electrofusion. The enucleated demicytoplasts were immersed in phytohemagglutinin (0.5 mg/mL in T2) for 3–4 s and then transferred to T2 containing donor cells at a low cell density. Each demicytoplast was then allowed to attach to a single, rounded, medium sized cell by gently rolling the demicytoplast over it. The couplets (demicytoplast-donor cell pairs) were transferred to fusion medium (0.3M D-mannitol, 0.1 mM MgCl_2_, 0.05 mM CaCl_2_ and 1 mg/mL polyvinyl alcohol) for 5 min equilibration. A single step fusion protocol was followed wherein a demicytoplast and a couplet were picked using a fine pulled capillary pipette (Unopette Becton Dickinson, NJ, USA) having an internal diameter of 100–150 μm. Initially, the couplet was expelled and aligned with an A.C. pulse (4 V) using BTX Electrocell Manipulator 200 (BTX, San Diego, CA, USA), so that the somatic cell faced the negative electrode, and immediately after alignment, another demicytoplast was introduced into the fusion chamber (BTX microslide 0.5mm gap, model 450; BTX, San Diego, CA) close to the somatic cell. As soon as the somatic cell was sandwiched between the demicytoplasts, single D.C. pulse (3.36 kV/cm for 4 s) was applied. The triplets were then incubated in T20 (for rounding up and subsequent reprogramming) for 6 h. The reconstructed oocytes were activated by incubating in T20 containing 4 μM calcimycin A23187 for 5 min at 38.5°C. After washing thrice with T20, the reconstructed oocytes were individually transferred to 5 μL droplets of T20 containing 2 mM of 6-dimethylamino purine, covered with mineral oil and incubated for 4 h in a CO_2_ incubator. The reconstructed, activated embryos were then transferred to 400 μL of RVCL medium containing 1% fatty acid-free-BSA in 4-well dishes (15–20 embryos/well), covered with mineral oil and kept undisturbed in a CO_2_ incubator for 8 days.

### Isolation, culture and cryopreservation of TE cells

Mitomycin C-inactivated buffalo fetal fibroblast (BFF) feeder layers were prepared as described earlier [27]. Day 8 IVF-derived hatched blastocysts and HMC-derived blastocysts were seeded on feeder layer as described earlier [8] with some modifications. Briefly, the blastocysts were gently pressed on the feeder layer with the help of 27 G hypodermic needle in 100 μL droplet of TE culture medium (DMEM + 10% FBS + 2 mM L-glutamine + 50 μg/mL gentamicin sulphate + 1% non-essential aminoacids + ITS (catalog no. I3146-5ML, Sigma) and were cultured in a CO_2_ incubator. The primary TE cell colonies appeared within 2 weeks as a tight monolayer of cuboidal cells. The outgrowths were mechanically dissected using a microblade for the separation of these cells from the ICM cells, and were subcultured in a 1:2 split ratio, which was maintained for the first 2–4 passages. The cells were further subcultured every 10–12 days, by mechanical dissociation, on fresh feeder layer in a split ratio of 1:4 till they remained alive in culture. The colony diameter and area were measured using a Nikon phase-contrast microscope (Eclipse Ti, Nikon, Tokyo, Japan) with NIS-Elements BR 3.1 software (Nikon, Tokyo, Japan).For cryopreservation, small clumps of TE cell colonies were suspended in 1 ml of freezing medium (DMEM + 10% dimethyl sulfoxide + 20% FBS) in Cryovials and were subjected to slow freezing up to -80°C following which these were transferred to liquid nitrogen.

### Characterization of TE cells

For examining their morphology, the cells were regularly observed under a phase-contrast microscope. Then, to assess the identity of our isolated cells, we set out to screen for the expression of a range of trophoblast markers by RT-PCR and immunostaining at regular intervals. *IFN-τ*, *CDX2*, *CYTOKERATIN-8* and *-18*, *GATA2*, *GATA3*, *ETS2*, *ELF-5*, *PAG2* and *FGFR-2* were examined by RT-PCR. Primers were designed using the internet-based software PRIMER-3 (http://www-genome.wi.mit.edu/cgi-bin/prime/primer3-www.cgi. Total RNA was isolated from TE cells and placenta using TRIzol (Invitrogen, Carlsbad, CA, USA) reagent according to manufacturer’s protocol. RNA integrity was checked by 1.5% agarose gel electrophoresis, which showed 2 bands of 28S and 18S. DNase treatment was done to remove genomic contamination using DNA-free kit (Ambion, Austin, TX, USA). The quality of RNA was checked by Nanoquant (Teccan, Salzburg, Austria). The RNA (1 μg) was reverse transcribed by Revertaid first strand cDNA synthesis kit (K1621, Thermo Scientific, Waltham, MA, USA), as per manufacturer’s protocol. The PCR reaction was performed in a thermal cycler (Bio-Rad, Hercules, CA, USA) using the following program: initial denaturation at 95°C for 3 min, followed by 95°C for 30 sec., annealing temperature for 30 sec, 72°C for 30 secs for 39 cycles and 72°C for 10 min in the last cycle. The annealing temperature and PCR conditions of the target genes are given in S1 Table.

The expression of CDX2 and cytoskeletal proteins (cytokeratin-18, keratin, vimentin and tubulin) was examined by immunofluorescence staining. TE cells that had been cultured in 96-well plates were fixed with methanol and then washed 3 times with DPBS and then permeabilized with 1% Triton X-100 in DPBS for 1h. After thorough washing with DPBS, the cells were incubated with the blocking solution (5% BSA) for 1h, followed by an overnight incubation at 4°C with the primary antibody which included anti-CDX2 (ready-to-use, AM392-10M, Bio-Genex Inc., San Ramon, CA, USA), anti-cytokeratin-18(1:200, sc-32329, SantaCruz Biotechnology, Dallas, TX, USA), anti-keratin(1:500, MAB1611, Milipore, Temecula, CA, USA), anti-vimentin (1:200, V6630, Sigma), anti-tubulin (1:400,T8328, Sigma). For negative controls, the entire procedure was followed except that the primary antibody was replaced with mouse IgG. After 3 washings with DPBS containing 0.1% Triton-X-100 (0.1% DPBST), the TE cells were incubated with the appropriate FITC-labeled secondary anti-mouse antibody (1:1000, F0257-.5ML, Sigma) for 1h. The cells were washed 3 times with 0.1% DPBST followed by nuclear staining with either Hoechst 33342or propidium iodide (PI). The cells were then examined under a fluorescence microscope (Diaphot, Nikon, Tokyo, Japan) after addition of antifade solution. Each experiment was repeated at least 3 times.

### BrdU cell proliferation assay

Cell proliferation was examined by incorporation of 5-bromo-2’-deoxyuridine-5’-triphospahate (BrdU, B5002-250MG, Sigma) into DNA of proliferating cells. BrdU label (10 μL of 10 mM solution) was added to each well of 96-well plate in which TE vesicles had been seeded and the plate was incubated for 24 h in a CO_2_ incubator at 37°C. The cells were fixed with chilled methanol at -20°C for 20 min and were permeabilized by treatment with 1% Triton-X-100 for 1 h. Following blocking of the cells by treatment with 5% BSA for 1 h, monoclonal anti-BrdU produced in mouse (B8434-200UL, Sigma) was added to each well except those for the negative control and the plate was incubated overnight at 4°C. The wells were then washed 3 times with 0.1% DPBST and were incubated with FITC-labeled anti-mouse IgG (1:1000, F0257-.5ML, Sigma) for 1 h. The wells were washed 3 times with DPBST after which the nuclear stain (PI) and antifade solution was added to each well. The cells were observed under a fluorescence microscope (Diaphot). Proliferation index = (number of BrdU positive nuclei/total number of nuclei counted in that field) × 100. Each experiment had 2 replicates and was repeated at least 3 times.

### TUNEL Assay

The level of apoptosis was determined by TUNEL staining using In Situ Cell Death Detection Kit, Fluorescein (11684795910, Roche) to assess the quality of day 8 blastocysts and TE cells cultured under different conditions. TE cells cultured in 96-well plate and day 8 blastocysts were fixed with 4% paraformaldehyde for 1 h and permeabilized by incubating with 0.5% Triton X-100 for 1 h. The cells and blastocysts were then incubated with FITC-conjugated dUTP and terminal deoxynucleotidyl transferase (TdT) enzyme for 1 h at 37°C in dark. The treated cells were added to RNase (50 μg/mL) and were stained with Hochest 33342 (5 μg/mL) for 5 min at 37°C in dark. For the positive control, the cells and blastocysts were treated with DNase solution (100 U/ml) for 20 min at 37°C prior to incubation with FITC-conjugated dUTP and TdT. The stained blastocysts were washed with DPBS (Ca^2+^ and Mg^2+^-free) and were mounted on glass slides in 3 μL droplets of antifade solution and were flattened with a cover slip. Cell counting was performed from the digital images obtained on inverted Nikon fluorescence microscope. Each experiment was repeated at least 3 times. Apoptotic index = (number of TUNEL positive nuclei/total number of nuclei counted in that field or blastocysts) × 100.

### Immunofluorescence staining for epigenetic markers

Because cloning is often associated with epigenetic errors, we aimed at determining the global level of some important histone marks in our cells/embryos. The global level of H3K18ac and H3K27me3 was compared in FF, AF and TE donor cells and in cloned blastocysts produced from these cells, using IVF blastocysts as controls, by immunofluorescence staining as described earlier [25] with some modifications. Briefly, the cells/embryos were fixed in 4% paraformaldehyde, permeabilized in 0.5% Triton X-100 and blocked in 5% BSA. These were then incubated overnight at 4°C with the respective rabbit primary antibody (anti-H3K18ac, 07–354, 1:1500, Millipore; anti-H3K27me3, ABE44, 1:1500, Millipore, MA, USA) diluted in the blocking reagent. For CDX2 staining, mouse primary anti-CDX2 antibody (ready-to-use, AM392-10M, Bio-Genex) was used. After washing 3 times with DPBS containing 0.1% Triton X-100 (DPBST), the blastocysts were incubated with FITC-conjugated goat anti-rabbit antibody (Sigma) for epigenetic markers and Alexa Fluor 594-conjugated donkey anti-mouse IgG (H+L) secondary antibody (A21203, Invitrogen) for CDX2, diluted 1:1000 in DPBS. After washing 5 times with DPBST, the nuclei were counterstained with Hochest 33342 (10 μg/ml) and rinsed with DPBST. The blastocysts were then mounted on slides in mounting medium (2.5% DABCO in glycerol). The slides were observed under a fluorescence microscope, and the images were captured keeping the same optical conditions. NIS-element basic research image processing software (Nikon, Tokyo, Japan) equipped with the microscope was used for image acquisition and quantitative measurements of the mean pixel intensity emitted by each individual nucleus. The mean pixel intensity for each blastocyst for each epigenetic marker was normalized by dividing with mean pixel intensity of Hoechst 33342 nuclei. The images were merged by NIS-Elements BR 3.0 software (Nikon, Tokyo, Japan). At least 10 blastocysts (60–80 nuclei/blastocyst) were analyzed for each epigenetic modification.

### Quantitative real-time PCR (qPCR)

Since cloned embryos have been reported to show aberrant gene expression, we then determined the relative expression level of some important genes by qPCR in different types of donor cells and in cloned embryos produced from them. RNA was isolated from blastocysts (n = 10 each) by RNAqueous micro kit (Ambion, Austin, TX, USA) or from TE cells using TRI Reagent (T9424-100ML, Sigma) as per manufacturers’ protocols. Following DNase treatment, RT reaction was performed for cDNA preparation using superscript reverse transcriptase III (Invitrogen). Quantification of mRNA was carried out by qPCR using CFX 96 I Cycler (Biorad). The reaction mixture (10 μL) contained 5 μL SYBR Green master-mix (Maxima SYBR Green Mastermix, Thermo Scientific), 0.2 μL of 10 μM of each primer and 2×diluted c-DNA. Thermal cycling conditions consisted of initial denaturation at 95°C for 5 min, followed by 40 cycles of 15 sec at 95°C, 15 sec at the corresponding annealing temperature and 15 sec at 72°C followed by 95°C for 10 sec (S1 Table). All primer pairs used were confirmed for their PCR efficiency, and specific products were checked by melt curve analysis and for the appropriateness of size by 2% agarose gel electrophoresis. Primer sequences are provided in the supplementary data (S1 Table). The expression data were normalized to the expression of GAPDH and were analyzed with CFX Manager software (BioRad). In all the experiments, three trials were carried out, each with duplicates.

### Preparations of fetal fibroblast-conditioned medium (CM)

For preparation of CM, BFF between passage 3 and 6 were cultured in DMEM/F12 supplemented with 10% FBS for 72 h in a CO_2_ incubator. The spent medium was collected, centrifuged at 1000xg for 10 min, filtered through 0.22 μm filters with PVDF membrane and was stored at -20°C until further use. Before use for TE culture, the CM was mixed with DMEM/F12 in a 1:1 ratio.

### Growth on collagen matrix and MaxGel ECM

Collagen matrix and MaxGel ECM were prepared as per the manufacturer’s (Sigma) protocol. Briefly, a mixture of 25 μL rat tail collagen Type I/mL of DMEM/F12 or 10 μl of MaxGel ECM/mL of DMEM was pipetted out in 48-well plates (400 μL/well), which were incubated for 2 h at 37°C in a CO_2_ incubator. The medium was removed after 2 h, and fresh TE culture medium was added before culture of TE cells.

### Experimental design and statistical analysis

#### Establishment of TE cells

Hatched blastocysts and blastocysts were produced through IVF and HMC, respectively. These were seeded on mitomycin C-inactivated BFF feeder layer and cultured in TE culture medium for comparing their attachment rate. The growth rate of primary colonies derived from the two types of blastocysts was then compared following culture for 7 days on feeder layer.

#### Culture under feeder-free conditions

Attempts were then made to develop a feeder-free system for the culture of TE cells. For this, TE cells from two sources were cultured in TE culture medium on MaxGel ECM-coated 48-well plates for 8–10 passages. For comparing the growth rate of the two types of TE cells, we then isolated TE cell vesicles of similar size (n = 3–4) obtained from the two types of TE cells, seeded them on MaxGel ECM-coated 48-well plates and cultured them in TE culture medium for 6 days. The next step was to find out the longevity of the TE cells derived from the two sources under feeder-free conditions for which we cultured them on MaxGel ECM-coated 48-well plates in TE culture medium for as long as they could survive in culture, after being passaged every 10–12 days.

#### Characterization of TE cells

For examining their morphology, the cells were regularly observed under a phase-contrast microscope. TE-IVF and TE-HMC cells were characterized at regular intervals by examination of the expression of TE-specific markers by RT-PCR and immunofluorescence. TE cells at passage 10, 20 and 30 were characterized by examining the expression of trophoblast-specific marker CDX2 by immunofluorescence staining. These cells were also characterized by examining the expression of cytoskeletal markers. Cell apoptosis and proliferation were measured by TUNEL and BrdU assay, respectively. In addition, the nuclear-to-cytoplasmic ratio of TE-IVF cells (passage 12–15) was compared with that of fibroblast cells. In this experiment, the TE-IVF cells were grown in a feeder-free environment to avoid contamination of TE cells with feeder fibroblasts.

#### Optimization of feeder-free culture

In order to optimize a feeder-free system for the in vitro culture of TE cells, TE cell vesicles of similar size (n = 3–4) at passage 8–10 were seeded in 48-well plates and were cultured for 7 days in TE culture medium following which the growth pattern, colony area, apoptotic index and proliferation index were measured in the following two experiments. For examining the effect of matrix on the growth of TE-IVF cells, these cells were divided into 4 groups and were cultured for 8 days in TE culture medium on the following surface. Group 1: BFF feeder layer (control); Group 2: uncoated plastic surface; Group 3: collagen-coated surface and Group 4: MaxGel ECM-coated surface. Then, the effects of fetal fibroblast CM on the colony area, apoptotic index and proliferation index were investigated for which, TE-IVF cells at passage 8–10 were divided into the following 2 groups and were cultured for 8 days in TE culture medium. Group 1: MaxGel ECM-coated surface and Group 2: MaxGel ECM-coated surface + fetal fibroblast CM.

#### Gene expression and epigenetic status of TE cells and HMC embryos derived from them

We then compared the expression level of *DNMT1*, *DNMT3a*, *GATA2*, *GATA3*, *ELF5*, *CDX2*, *ETS2*, *PAG1* and *PAG2* between TE-IVF and TE-HMC at passage 20–22, which had been cultured in TE culture medium on MaxGel ECM. Also, the expression level of *DNMT1*, *DNMT3a*, *CDX2*, *ETS2* and *PAG2* genes was compared between IVF hatched blastocysts and HMC blastocysts. Then we investigated the effect of FGF2 on the expression level of some trophoblast-specific genes in TE cells. TE-IVF cell vesicles of similar size (n = 4) at passage 25–28 were seeded in MaxGel ECM-coated 4-well plates and were cultured in TE culture medium for 7 days. The medium was then removed and replaced with TE culture medium lacking FBS for 24 h. Subsequently, this medium was also removed and was replaced with serum-free TE culture medium containing 0, 5, 20, 100 ng/ml of FGF-2 and the cells were cultured for 48 h. After this, the spent medium was collected and stored at -80°C until further analysis. Cells were processed for RNA isolation after which expression levels of trophoblast-specific genes *IFN-τ*, *CDX2*, *FGFR2*, *GATA2* and *GATA3* were examined by qPCR.

HMC embryos were then produced using three different types of donor cells i.e., FF, AF (passage 5–15) and TE donor cells (passage 5–25). Their developmental competence was compared by recording the cleavage and blastocyst rate. The blastocyst quality was compared by determining the total cell number (TCN) and the apoptotic index by TUNEL assay. Then, the global level of H3K27me3 and H3K18ac was compared in FF, AF and TE donor cells and in cloned blastocysts produced from these cells, using IVF blastocysts as controls. The *in situ* expression of CDX2 and the relative mRNA abundance of *OCT4*, *SOX2*, *IFN-τ*, *CDX2*, *DNMT1*, *DNMT3a*, *GATA2* and *GATA3* were also compared among cloned blastocysts derived from FF, AF and TE donor cells, using IVF blastocysts as controls.

Statistical analysis was carried out using Sigma Stat version 3.1 (Aspire Software International, VA, USA). The datasets were analyzed by one-way analysis of variance (ANOVA) followed by Holm-Sidak test. Percentage values were subjected to arcsine transformation prior to analysis. The differences were considered to be statistically significant at *P<*0.05. Data were presented as mean ± SEM.

## Results

### Establishment of TE cells

**Fig 1:**
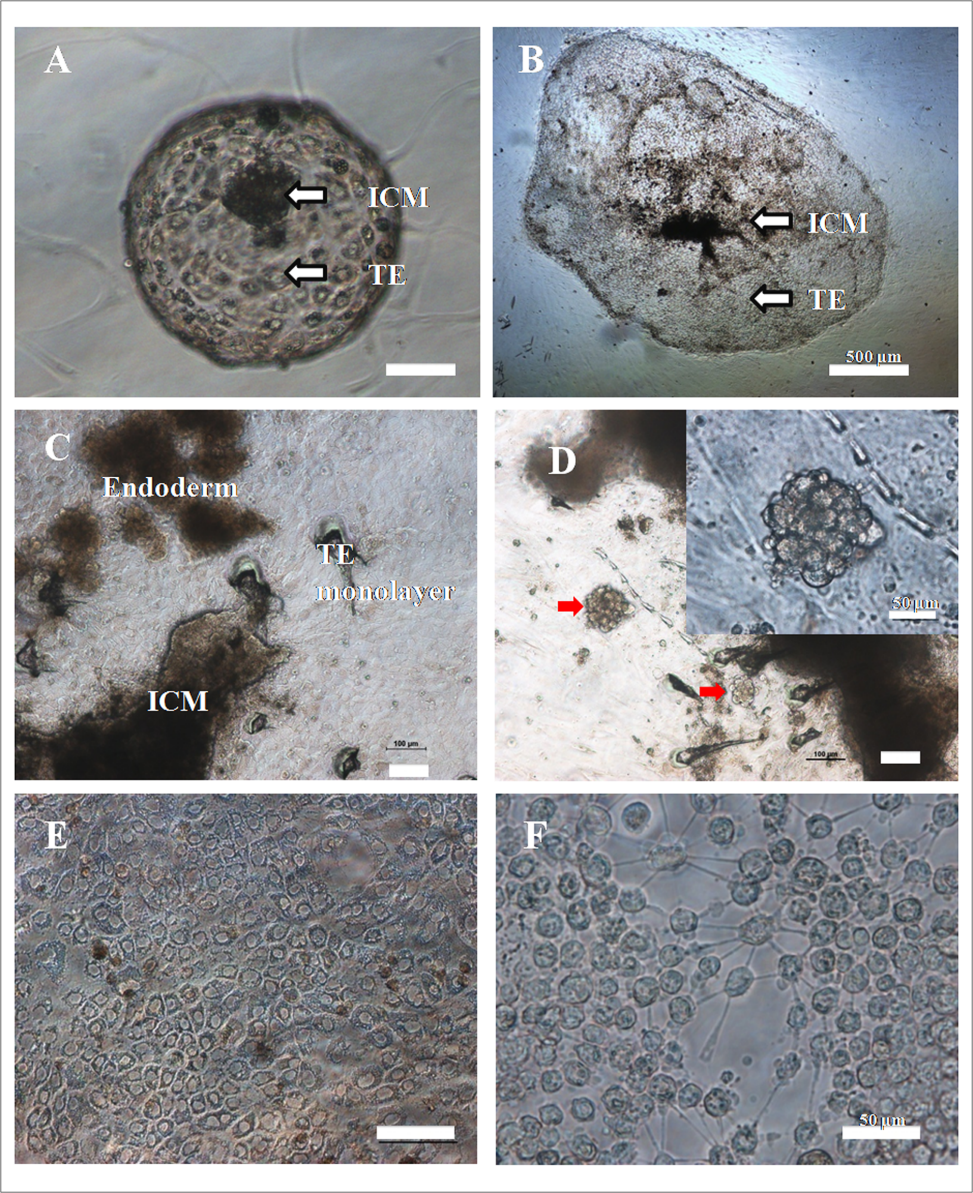
Derivation of presumptive TE cells. (A) An IVF-derived blastocyst seeded on buffalo fetal fibroblast feeder layer; (B) A primary colony of TE cells (40X, Scale bar = 500 μm); (C) A primary colony showing inner cell mass and endoderm cells (100X); (D) Endoderm colonies indicated by the arrow mark showing tight colony morphology (100X), Inset: Endoderm colony at 400X (Scale bar = 50 μm); (E) TE cells at passage 20 (200X) and (F) TE cells digested with accutase showing loosening of cells and thread-like structures which are the parts of tight junctions (400X, Scale bar = 50 μm). Scale bar = 100 μm, unless otherwise mentioned in Figure.

**Fig 2:**
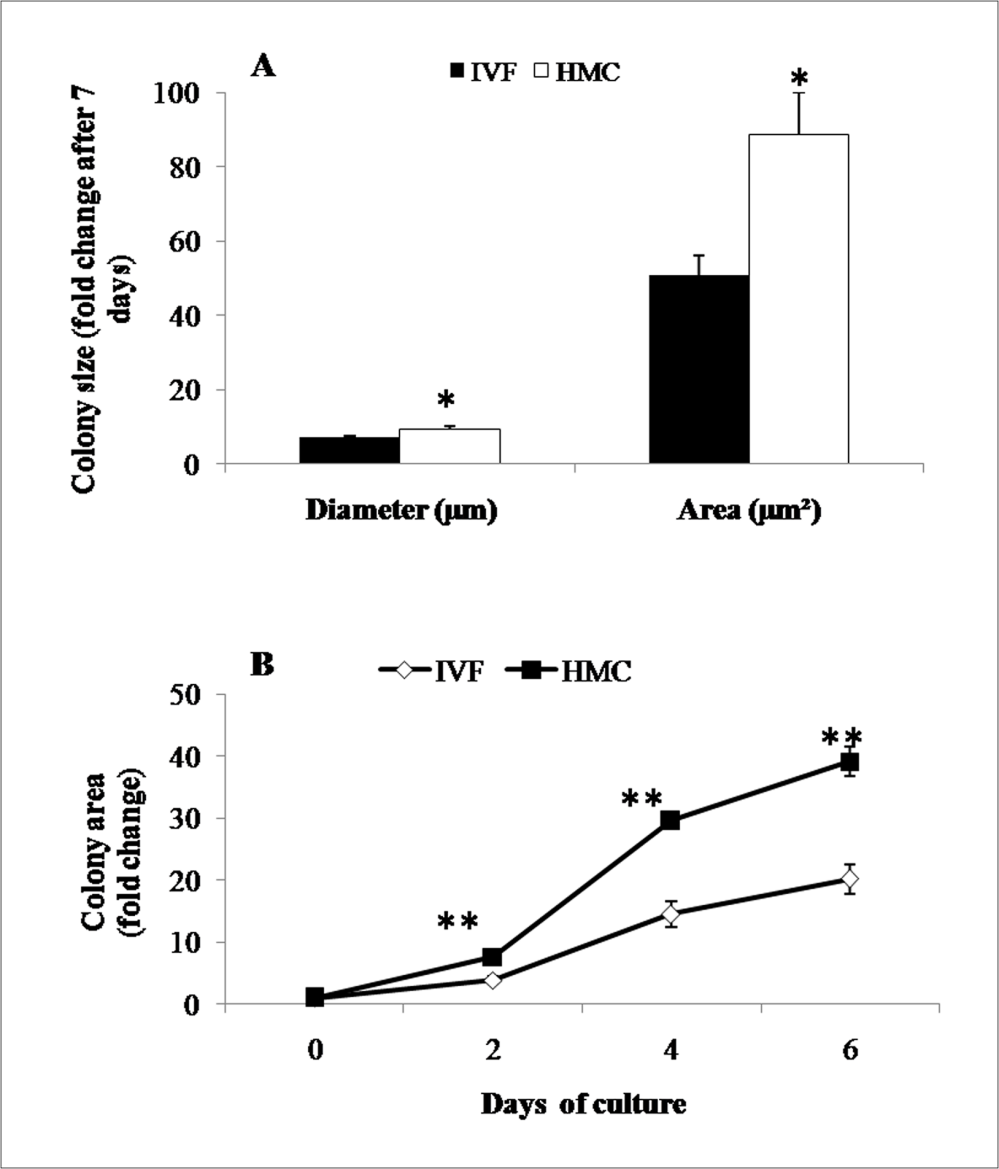
Growth analysis of presumptive TE cells from different sources. Growth rate of (A) primary colonies on fetal fibroblast feeder layer and (B) TE cells obtained from IVF-derived hatched blastocysts and HMC-derived cloned blastocysts on MaxGel ECM under feeder-free conditions. Bars/graph points marked with an asterisk differ significantly from the corresponding value. *(P<0.01); **(P<0.001).

In the first experiment, in which IVF-derived hatched blastocysts and HMC-derived blastocysts were seeded on mitomycin C-inactivated BFF feeder layer, the attachment rate of former (41/63, 65%)was significantly (P<0.05) higher than that of latter (17/35, 48.6%). Primary colonies of presumptive TE cells appeared after 2 weeks, and had flat, epithelial, sheet-like morphology (Fig 1). In addition to presumptive TE cell colonies, epiblast-derived fibroblasts were also observed (S1 Fig). We then compared the growth rate of primary colonies obtained from the two types of blastocysts within the first 7 days of seeding on feeder layer. It was found to be lower (P<0.01) for IVF- than that for HMC-derived blastocysts (Fig 2A).

### Culture under feeder-free conditions

In our study aimed at developing a feeder-free system for the culture of presumptive TE cells, we were successfully able to culture IVF-derived trophectoderm (TE-IVF) and HMC-derived trophectoderm (TE-HMC) cells in TE culture medium on MaxGel ECM-coated 48-well plates. When these cultures had reached 8–10 passages (each passage at an interval of 10–12 days), we compared their growth rate by isolating TE cell vesicles of similar size (n = 3–4) from the two types of presumptive TE cells, and culturing them on MaxGel ECM-coated 48-well plates in TE culture medium for 6 days. We found that the growth rate of TE-HMC cells was higher (P<0.001) than that of TE-IVF cells (Fig 2B).

Next, we aimed at assessing the longevity of the presumptive TE cells derived from the two sources under feeder-free conditions. We found that following culture on MaxGel ECM-coated 48-well plates in TE culture medium, the percentage of presumptive TE cell colonies which were able to survive the first subculture was higher (P<0.05) for TE-IVF cells (21/41, 51.2%) than that for TE-HMC cells (5/17, 29.4%). The number of cell lines that survived up to passage 5, 10 and 20, which was 7/41, 4/41 and1/41 (17.1, 9.8 and 2.4%), respectively, for TE-IVF cells was not significantly different from 3/17, 2/17 and 1/17 (17.6, 11.8 and 5.9%), respectively, for TE-HMC cells. TE-IVF and TE-HMC cell lines could survive for 41 (>450 days) and 36 passages (>390 days), respectively.

### Characterization of presumptive TE cells

**Fig 3:**
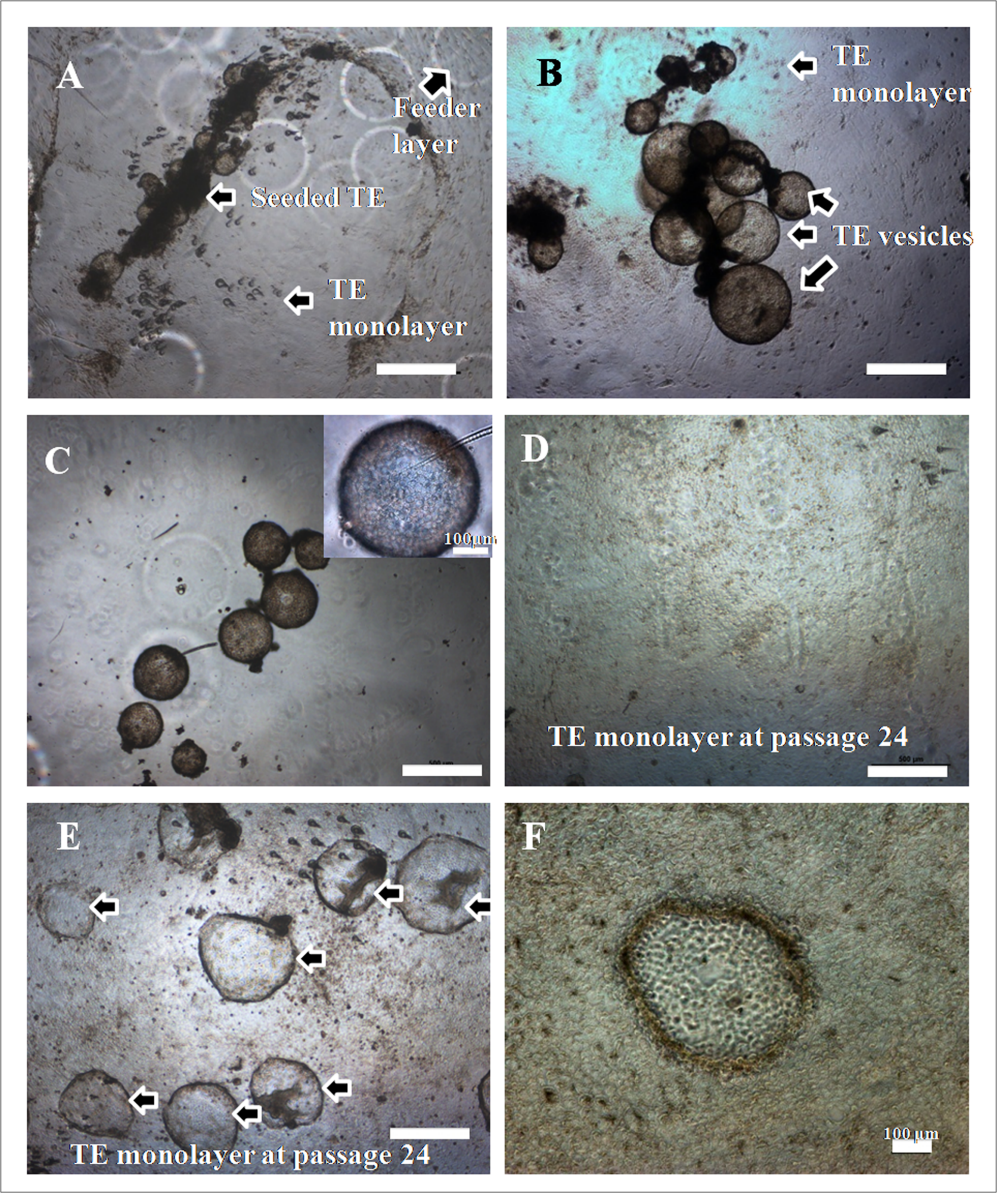
Morphology of presumptive TE cells under feeder and feeder-free conditions. Growth of IVF-derived trophoblast cells under (A) feeder and (B) feeder-free conditions. Subculture of trophoblast cells under feeder-free conditions led to development of vesicles of 100–500 μm diameter, which get dissociated (C). Following subculture, the trophoblast cells formed confluent monolayer on feeder layer (D) and dome shaped colonies under feeder-free conditions (E). Scale bar = 500 μm. Single dome observed under 100X (F). Scale bar = 100 μm.

The morphology of trophoblast vesicles and colonies produced under feeder- and feeder-free conditions is presented in Fig 3. The presumptive TE cell colonies were flat in appearance. The cells were polygonal in shape and had granular cytoplasm containing numerous lipid droplets with a centrally-placed large prominent nucleus. The diameter and area of the cells and their nuclei and the nucleus-to-cytoplasm was found to be higher (P<0.05) for presumptive TE cells than that of fibroblast cells (Table 1).

**Table 1:** Nuclear to cytoplasmic ratio in buffalo TE cells and fetal fibroblasts.

Dimensions	Cell type	Cell	Nucleus	Nucleus/cell ratio
Diameter (μm)	TE	18.7 ± 0.30^a^	14.8 ± 0.31^a^	0.78^a^
	FF	22.0± 0.20^b^	9.9 ± 0.10^b^	0.45^b^
Area (μm^2^)	TE	290.9 ± 9.68^x^	186.4 ± 7.72^x^	0.64^x^
	FF	385.6 ± 7.45^y^	79.3 ± 1.67^y^	0.20^y^

TE: TE cell; FF: Fetal fibroblast. Data from 3 trials. Data are Mean ± SEM of 200 cell/nuclei. Values with different superscripts (a vs. b or x vs. y) within the same column differ significantly (P<0.05).

**Fig 4:**
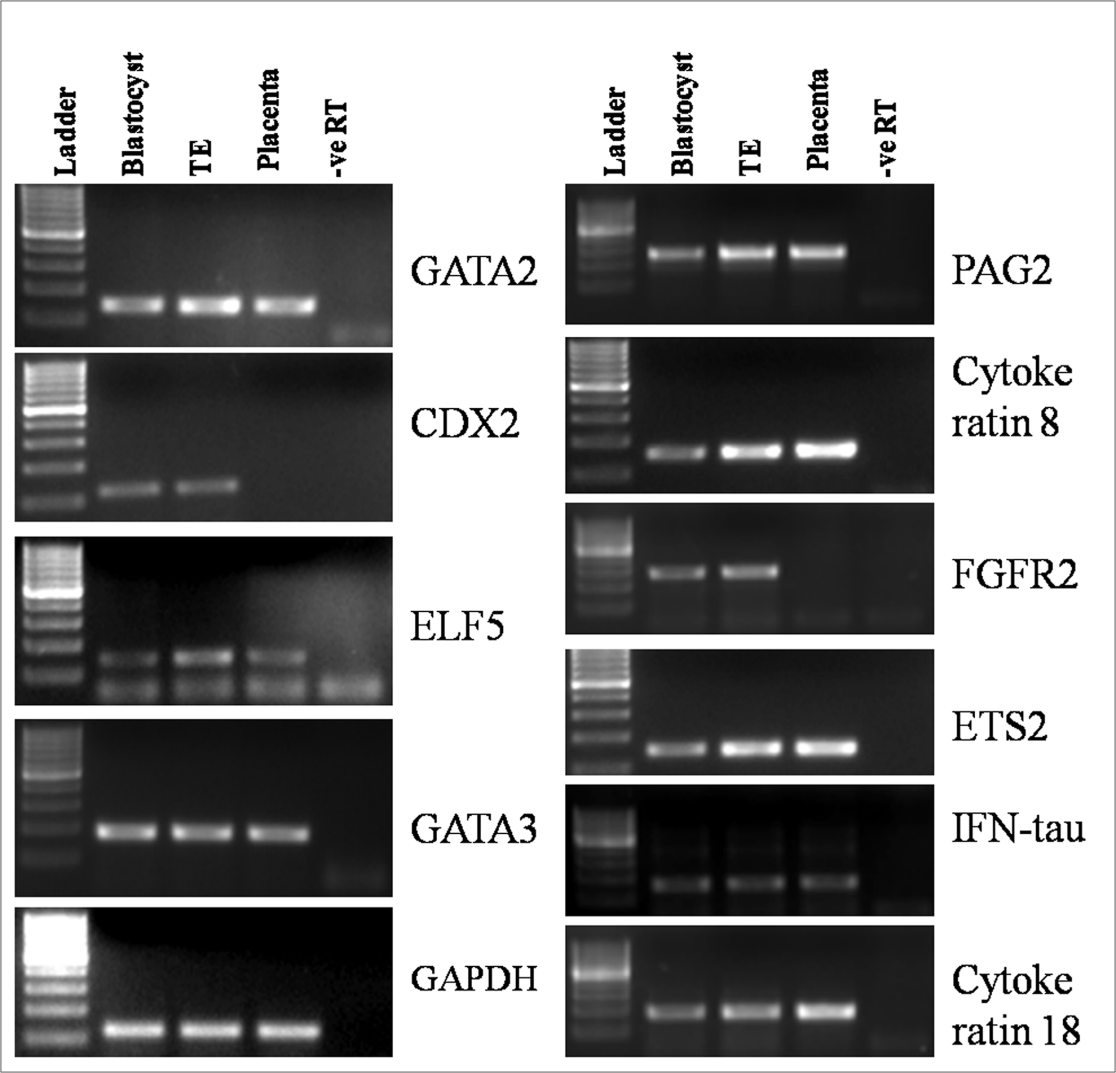
Characterization of presumptive TE cells by RT-PCR. Expression of TE-specific markers in blastocysts, trophoblast (TE) cells, and placental cells by RT-PCR.

**Fig 5:**
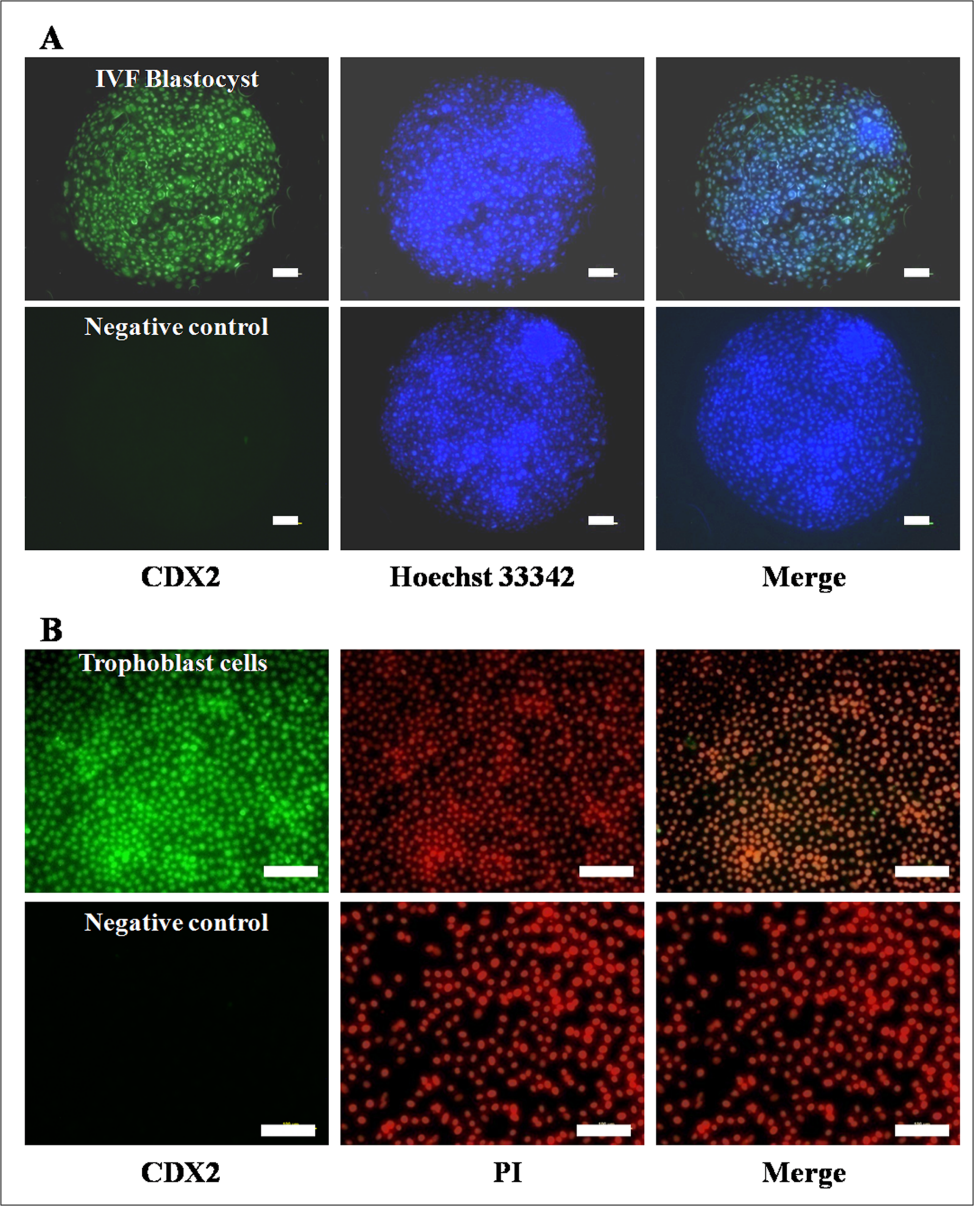
Immunostaining of CDX2. (A) IVF-derived hatched blastocysts (100X) and (B) TE cells produced under feeder-free conditions showing positive expression of CDX2 (200X). Scale bar = 100 μm.

**Fig 6:**
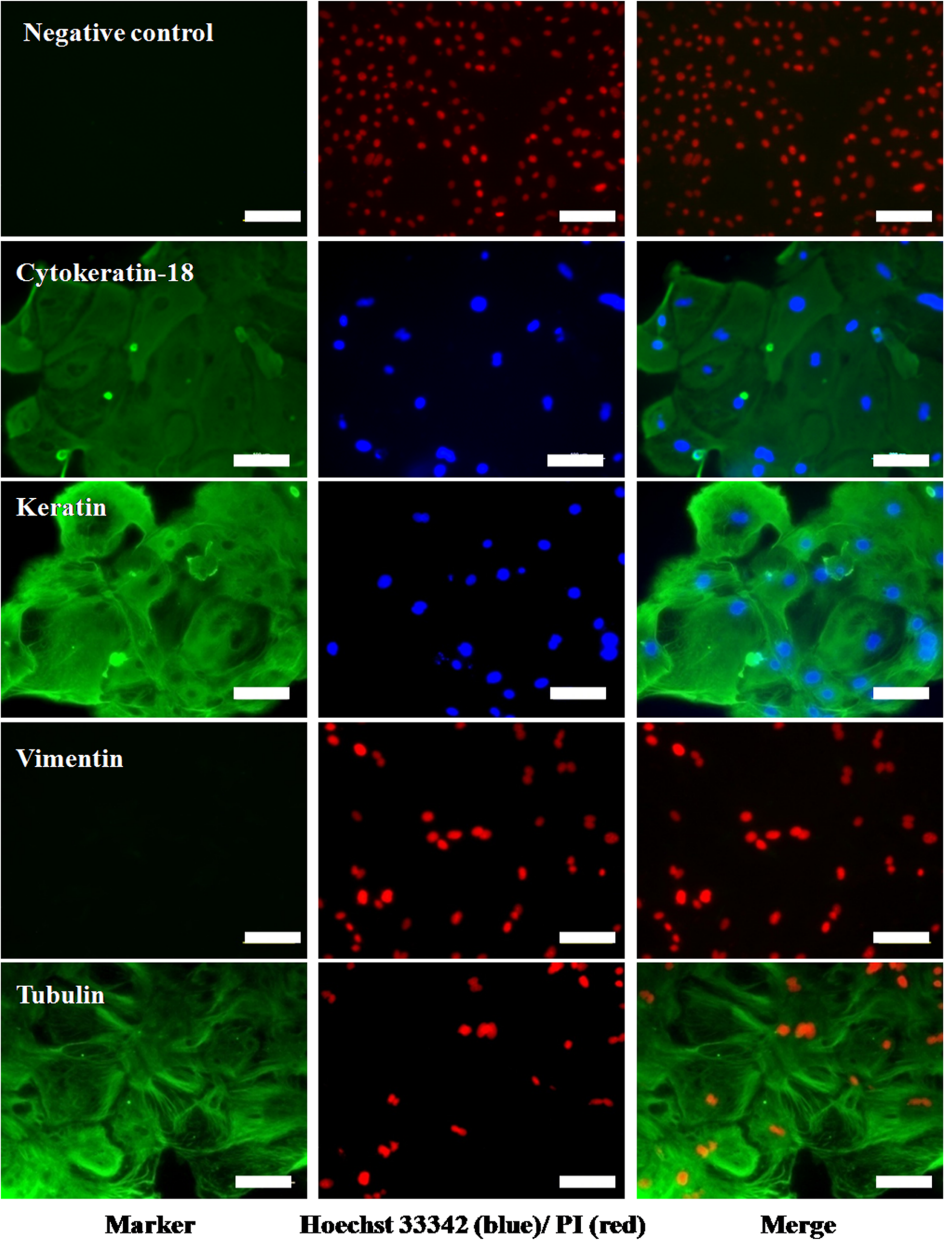
Characterization of presumptive TE cells by immunofluorescence for cytoskeletal proteins. TE cells produced under feeder-free conditions showing the expression of cytokeratin-18, keratin and tubulin but not that of vimentin, by immunofluorescence staining.

The presumptive TE cells were found to express TE-specific markers *IFN-τ*, *CDX2*, *CYTOKERATIN-8* and *18*, *GATA2*, *GATA3*, *ETS2*, *ELF-5*, *PAG2* and *FGFR-2* by RT-PCR (Fig 4). All these markers were found to be expressed in the blastocysts also. The expression of *CYTOKERATIN-8* and *-18* was present in buffalo mammary epithelial cells which were taken as their positive control (Data not shown). In comparison, placental cells showed expression of *PAG2*, *CYTOKERATIN-8* and *18*, *GATA2*, *GATA3*, *ETS2* and *ELF-5* but not that of *CDX2* and *FGFR-2*. The expression of CDX2 was confirmed to be present in blastocysts and TE cells by immunofluorescence staining (Fig 5). The presumptive TE cells and BFF feeder layer were characterized by examining the expression of vimentin by immunofluorescence staining. Following immunofluorescence staining, the presumptive TE cells were found to exhibit the expression of cytokeratin-18, keratin and tubulin but not that of vimentin (Fig 6).

### Optimization of feeder-free culture

In the experiment aimed at comparing the effect of matrix, the colony area on Day 7 of culture was found to be similar for cells grown on BFF feeder layer or dishes coated with collagen or MaxGel ECM, which was significantly higher (P<0.5) than that of cells grown on uncoated plastic surface (Table 2). The apoptotic index was significantly (P<0.05) lower for cells grown on feeder layer or MaxGel ECM that for those grown on uncoated plastic surface. The proliferation index was significantly (P<0.05) higher for cells grown on feeder layer than for those grown on collagen or MaxGel ECM coated dishes which, in turn, was higher (P<0.05) than that for cells grown on uncoated plastic surface. In the experiment focused on examining the effects of conditioned medium, fetal fibroblast CM was found to have no effect on the colony area and apoptotic index when the TE cells were cultured on MaxGel ECM-coated dishes (Table 3). However, the proliferation index was significantly (P<0.05) higher in the presence of CM than when it was absent.

**Table 2:** Effect of matrix on growth of TE cells.

Matrix	Colony area (mm^2^) on Day 7	Apoptotic Index	Proliferation Index
Fetal fibroblast feeder layer (control)	7.22 ± 0.72^b^	0.92 ± 0.12^b^	62.88 ± 2.92^c^
Plastic	3.93 ± 0.67^a^	1.83 ± 0.31^a^	35.44 ± 2.20^a^
Collagen	7.09 ± 0.85^b^	1.59 ± 0.04^ab^	44.74 ± 3.95^b^
MaxGel ECM	9.28± 1.26^b^	0.76 ± 0.18^b^	48.89 ± 1.67^b^

Data from 5 trials. Data are Mean ± SEM. Values with different superscripts (a, b and c) within the same column differ significantly (P<0.05).

**Table 3:** Effect of fetal fibroblast CM on growth of TE cells.

Matrix	Colony area (mm^2^) on Day 7	Apoptotic Index	Proliferation Index
MaxGel ECM	8.04 ± 1.36	0.76 ± 0.18	48.89 ± 1.67^a^
MaxGel ECM + CM	7.19 ± 1.35	0.54 ± 0.20	59.50 ± 4.25^b^

Data from 3 trials. Data are Mean ± SEM. Values with different superscripts (a and b) within the same column differ significantly (P<0.05).

### Expression level of genes in IVF and HMC blastocysts and in TE cells derived from them

**Fig 7:**
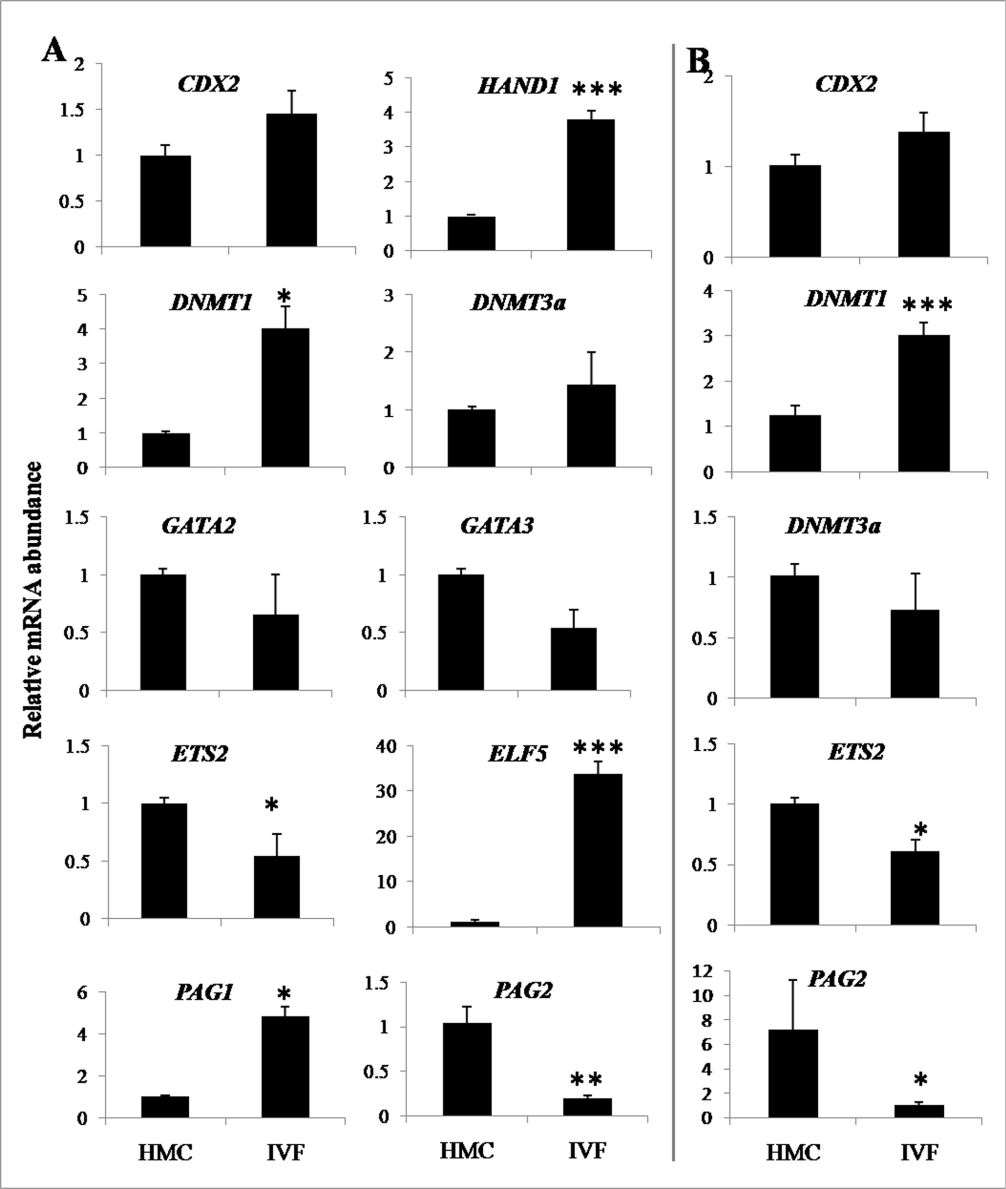
Relative mRNA abundance of some important genes. Relative expression of some important genes in (A) TE cells produced from HMC-derived cloned blastocysts and IVF-derived hatched blastocysts and in (B) blastocysts of the two types. Bars marked with an asterisk differ significantly from the corresponding value. * (P<0.05); ** (P<0.01); *** (P<0.001).

The relative mRNA abundance of *DNMT1*, *HAND1*, *ELF5* and *PAG1* was found to be higher (P<0.05 to P<0.001) and that of *ETS2* and *PAG2* to be lower (P<0.05 to P<0.001) in TE-IVF than in TE-HMC cells (Fig 7). The expression level of *DNMT3a*, *CDX2*, *GATA2* and *GATA3* was not significantly different between the two groups. The pattern of expression level of most of these genes in IVF and HMC blastocysts reflected that present in the TE derived from them. Whereas the expression level of *DNMT1* was higher (P<0.001) and that of *ETS2* and *PAG2* was lower (P<0.05 to P<0.001) in IVF than that in HMC blastocysts, the expression level of *CDX2* and *DNMT3a* was similar between the two types of embryos.

### Effect of FGF2 on expression level of some trophoblast-specific genes in TE cells

**Fig 8:**
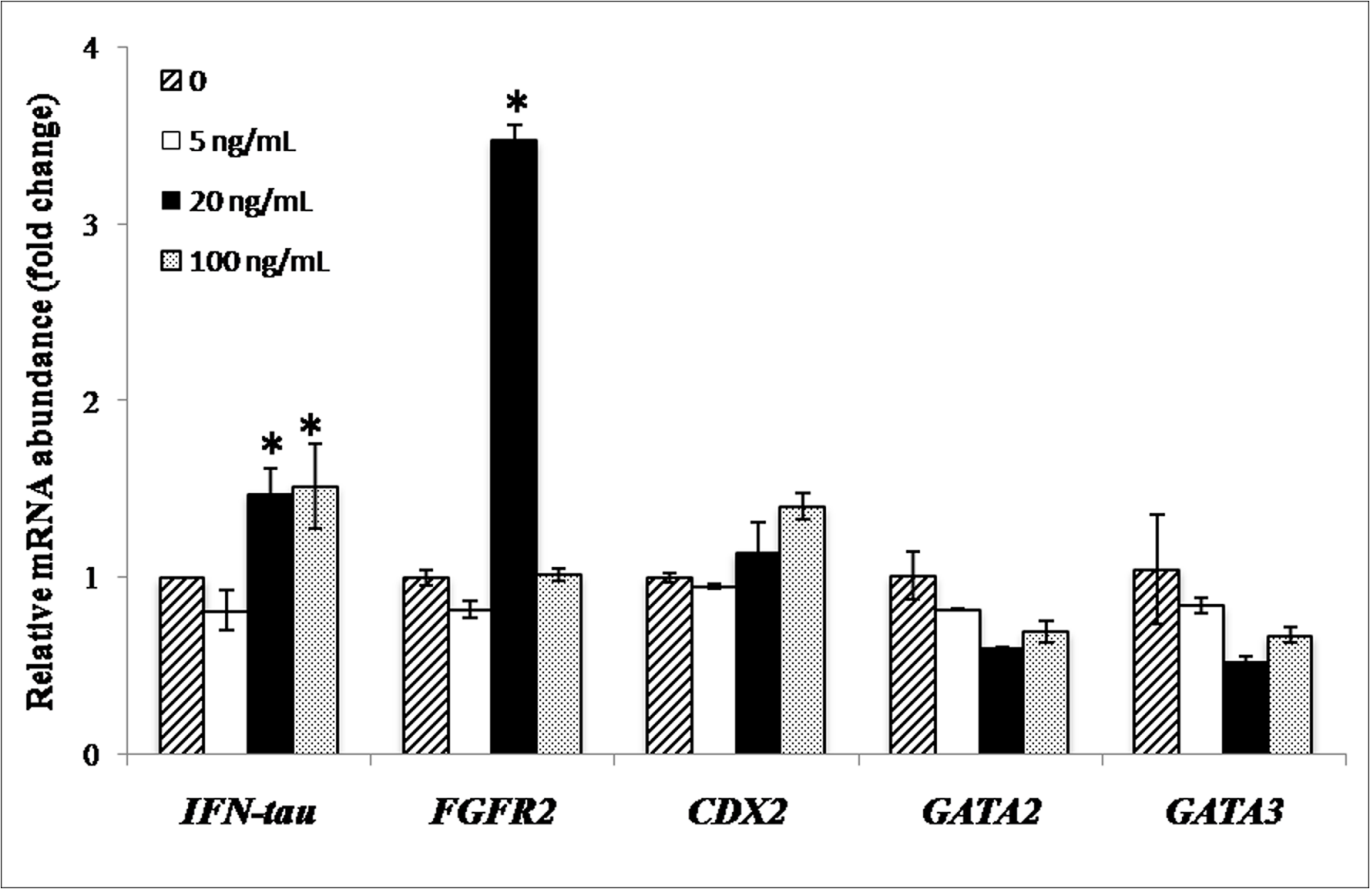
Effect of FGF2 on expression level of trophoblast-specific genes. Bars with different superscripts differ significantly (P<0.05).

Following culture of TE-IVF cells for 48 h in the presence of 0, 5, 20 or 100 ng/mL FGF-2, the relative mRNA abundance of *IFN-τ* was found to be higher (P<0.05) following supplementation with 20 or 100 ng/mL FGF2 than with 0 (control) or 5 ng/mL FGF2 (Fig 8). The expression level of *FGFR2* was higher (P<0.001) with 20 ng/mL FGF2 than that in the other groups whereas there was no effect of FGF2 on the relative transcript level of *GATA2*, *GATA3* and *CDX2*.

### Developmental competence and quality of HMC embryos produced using different types of donor cells

The cleavage rate of HMC embryos derived from TE cells was lower (P<0.05) than that of those derived from FF or AF (Table 4). The blastocyst rate was lower (P<0.05) for TE cells than that for FF or AF. The TCN of TE-derived blastocysts was lower (P<0.05) than that of FF-or AF-derived blastocysts which, in turn, was lower (P<0.05) than that for IVF blastocysts. The apoptotic index was higher (P<0.05) for TE- or AF- than that for FF-derived or IVF blastocysts.

**Table 4:** Developmental competence and quality of IVF and HMC embryos produced using different types of donor cells.

Blastocyst type	Donor cell type	Reconstructs (n)	Cleavage n (%)	Blastocysts n (%)	Total cell number	Apoptotic index
IVF	-	-	-	-	196.2 ± 8.45^a^	2.94 ± 0.30^a^
HMC	Adult fibroblasts	173	157 (90.63±2.73)^a^	69 (40.35±2.30)^a^	226.4 ± 13.74^b^	4.30 ± 0.40^b^
	Fetal fibroblasts	209	194 (92.49±1.64)^a^	97 (46.03±1.65)^b^	237.0 ± 15.54^b^	2.68 ± 0.43^a^
	Trophoblast cells	211	170 (79.81±1.48)^b^	44 (20.71±2.21)^c^	141.8 ± 11.46^c^	5.94 ± 1.16^b^

Data from 9 trials. Values are mean ± SEM. Values with different superscripts (a, b and c) within the same column differ significantly (P<0.05).

### Epigenetic status of FF, AF and TE cells and of cloned embryos produced from them

**Fig 9:**
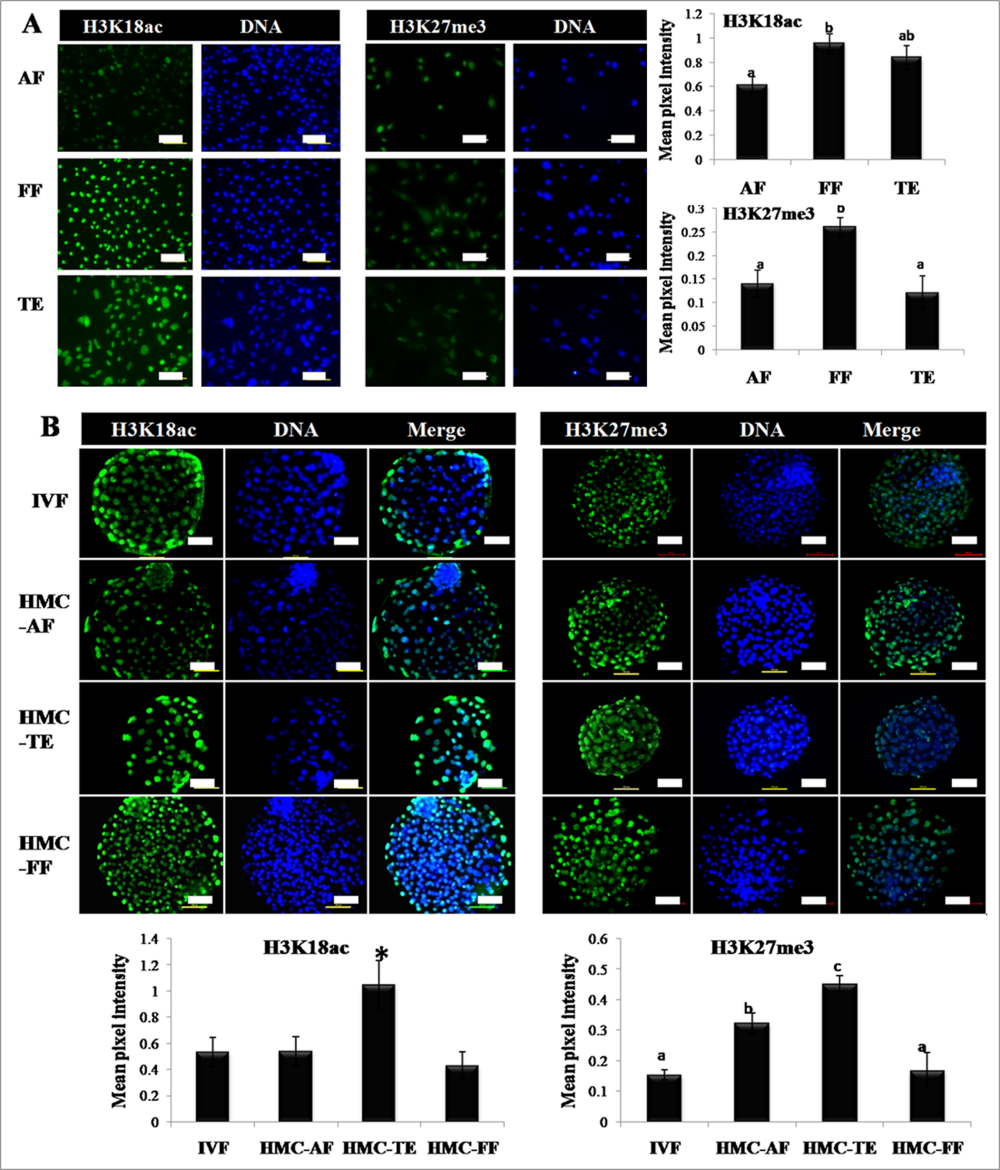
Global H3K18ac and H3K27me3 levels in different types of donor cells and embryos derived from them. (A) Global level of H3K18ac and H3K27me3 in trophoblast cells, adult fibroblasts and fetal fibroblasts. (B) Global level of H3K18ac and H3K27me3 in cloned embryos produced using trophoblast cells, adult fibroblasts and fetal fibroblasts as donor cells and those produced by IVF. Bars marked with an asterisk or different superscripts differ significantly from corresponding values (P*<*0.05).

**Fig 10:**
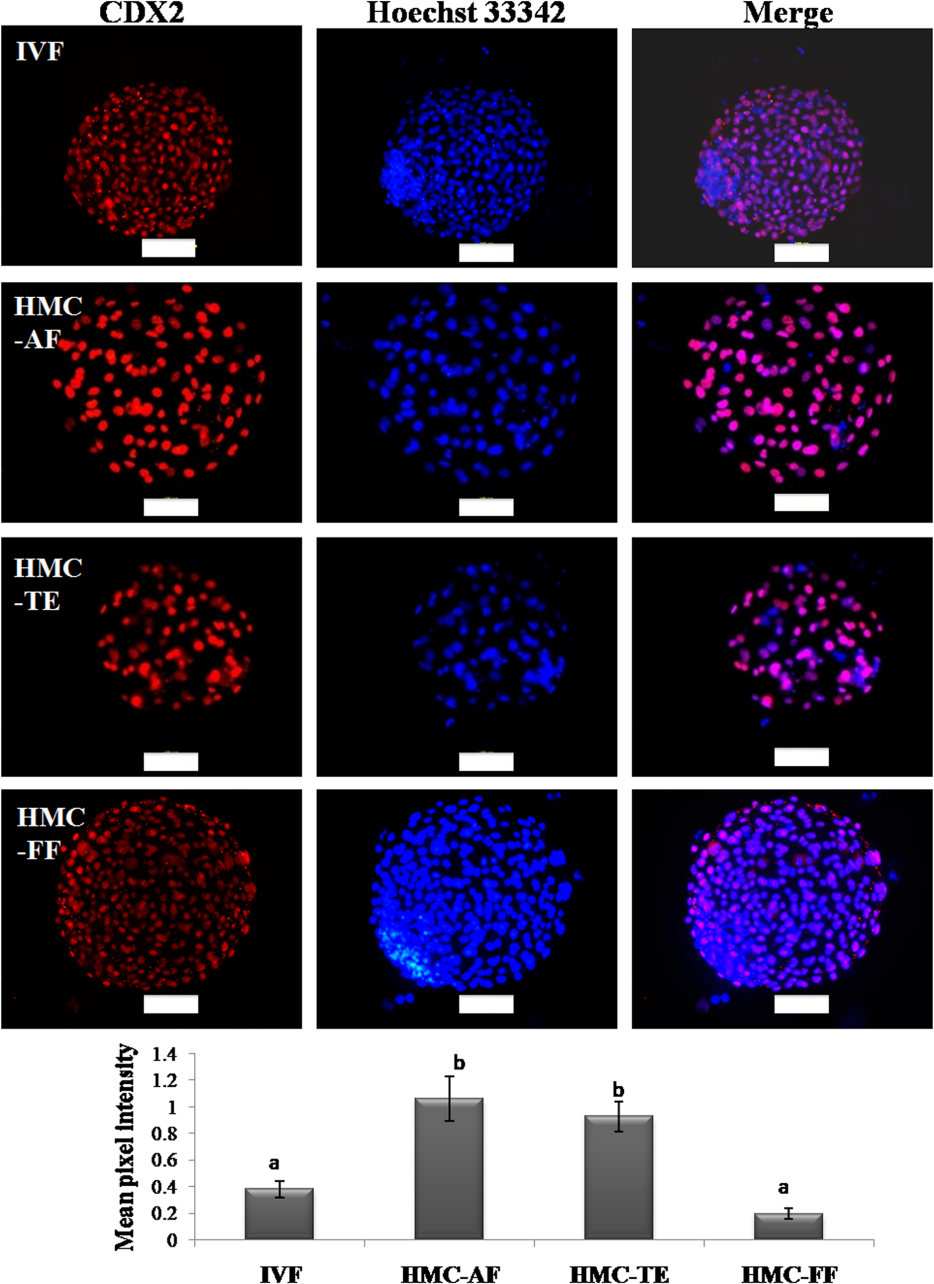
Global CDX2 levels in different types of embryos. Global level of CDX2 in cloned embryos produced using trophoblast cells, adult fibroblasts and fetal fibroblasts as donor cells and those produced by IVF. Bars marked with an asterisk differ significantly from corresponding values (P*<*0.05).

Global levels of H3K18ac and H3K27me3 were measured in different types of donor cells (Fig 9A) and embryos derived from them (Fig 9B). The global level of H3K27me3 was found to be higher (P<0.05) in FF than in AF or TE cells. The global level of H3K27me3 was higher (P<0.05) in TE- than that in AF-derived blastocysts which, in turn, was higher (P<0.05) than that in FF-derived or that in IVF blastocysts. The global level of H3K18ac was higher (P<.05) in FF than in AF cells whereas that of TE cells was not significantly different from the two. The global level of H3K18ac was higher (P<0.05) TE- than that in FF- or AF-derived or that in IVF blastocysts. *In situ* expression of CDX2 was higher (P<0.05) in TE- or AF- than that in FF-derived or IVF blastocysts (Fig 10).

### Expression level of genes in cloned blastocysts produced using FF, AF and TE cells

**Fig 11:**
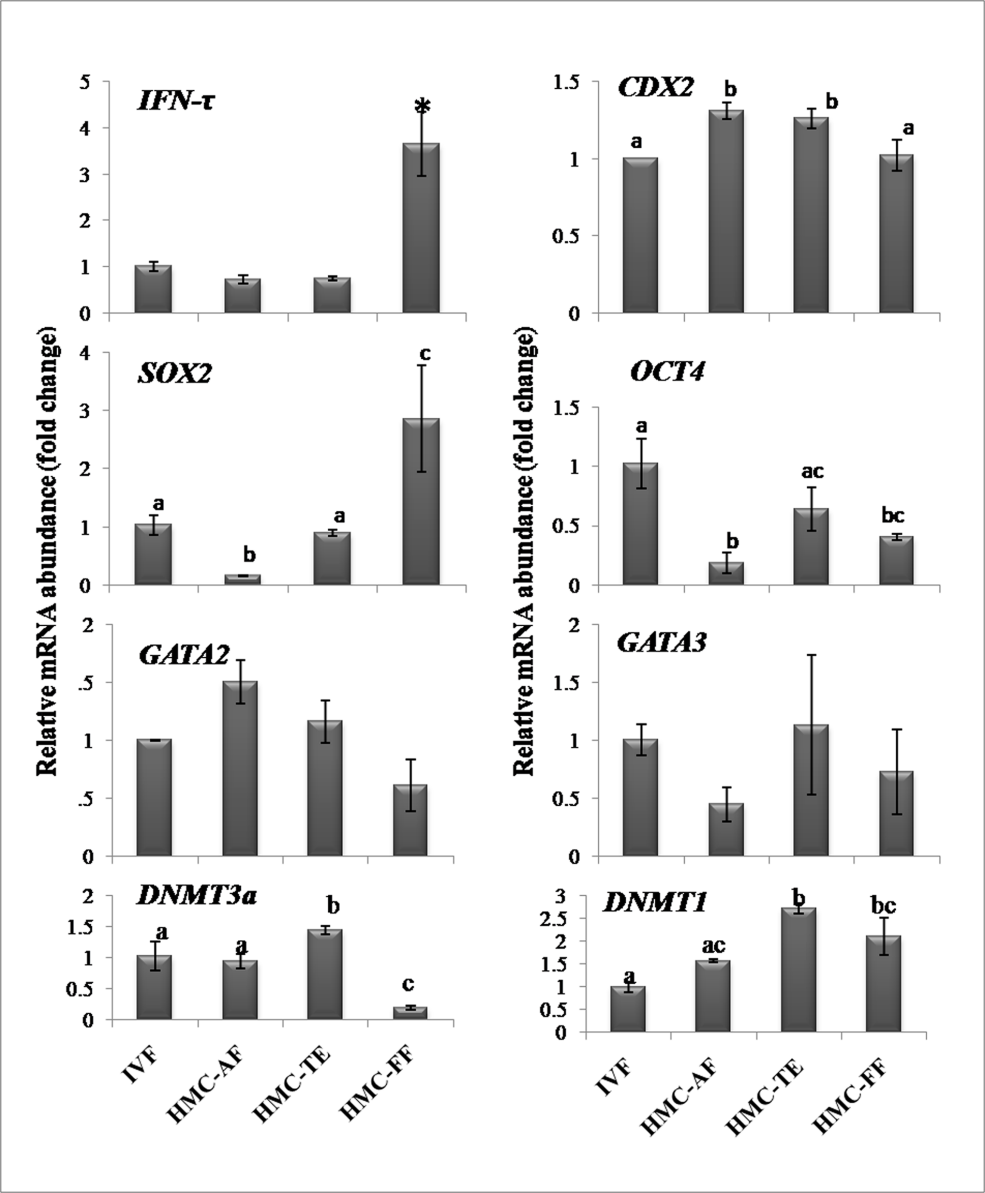
Relative mRNA abundance of some important genes in different types of embryos. Expression level of epigenetics- (*DNMT1* and *DNMT3a*), trophectoderm- (*IFN-tau*, *CDX2*, *GATA2* and *GATA3*) and pluripotency-related (*OCT4* and *SOX2*) genes in cloned embryos produced using trophoblast cells, adult fibroblasts and fetal fibroblasts as donor cells and those produced by IVF. Bars with different superscripts differ significantly (P*<*0.05).

Among epigenetics-related genes, the relative transcript level of *DNMT1* and *DNMT3a* was found to be higher (P<0.05) in TE- than that in AF-derived or IVF blastocysts (Fig 11). The expression level of *DNMT3a* was higher (P<0.05) in AF- than that in FF-derived blastocysts whereas that of *DNMT1* was not significantly different between the two groups. Among placenta-linked genes, the expression level of *CDX2* was higher in TE- or AF- than that in FF-derived or IVF blastocysts whereas that of *GATA2* and *GATA3* was not significantly different among the four groups. The expression level of *IFN-τ* was higher (P<0.05) in FF-derived than that in blastocysts derived from other cell types or that in IVF blastocysts. Among pluripotency-related genes, the transcript level of *OCT4* was higher (P<0.05) in TE-derived and IVF blastocysts than that in FF- or AF-derived blastocysts. The expression level of *SOX2* in the blastocysts was higher (P<0.05) in the order FF >TE cells and IVF>AF.

## Discussion

TE cell lines derived from NT embryos can be used as a research tool to investigate epigenetic deficiencies, abnormalities in the expression of important genes, IFN-τ production and placental growth disorders associated with NT embryos. In the first part of the study, we isolated and characterized buffalo TE cells derived from blastocysts produced by HMC. Also, the growth characteristics and gene expression were compared between TE-HMC and TE-IVF cells, and a feeder-free system was developed for their long-term in vitro culture. In the second part of the study, we used TE-IVF cells as donor cells to produce HMC embryos following which their developmental competence, quality, epigenetic status and gene expression were compared with those of HMC embryos produced using FF or AF as donor cells.

We found the primary colony formation rate to be higher with HMC-than that with IVF-derived blastocysts unlike others [18] which found it to be similar in bovine NT and IVF blastocysts. Our results suggest that TE-HMC cells have capability to grow in culture similar to that of TE-IVF cells as indicated by a higher growth rate of TE-HMC than that of TE-IVF cells and similar survival rate of the two types of TE cells during culture under feeder-free conditions. TE cell culture systems that require feeder cells for their maintenance have been developed in several species. These systems used feeder cells like mouse embryonic fibroblasts for bovine [22] and STO cells for bovine [8,17,18] and pig [11] TE cells. The development of a feeder-free culture system is necessary for investigating the characteristics of TE cells without the interference of contaminating feeder cells. In order to develop a feeder-free culture system for buffalo TE cells, we used attachment factors and supplementation with fetal fibroblast CM. We found that MaxGel ECM was superior to collagen-coated or uncoated dishes in terms of supporting cell proliferation and limiting apoptosis. Its ability to support the maintenance of TE cells was further confirmed by long-term culture of TE-IVF and TE-HMC cell lines, which could survive for 41 (>450 days) and 36 passages (>390 days), respectively under feeder-free conditions. A feeder-free culture system based on collagen-coated dishes and fibroblast CM that could support the maintenance of bovine TE cells for more than 18 months was reported by Shimada et al. [9]. These authors reported that fibroblast-CM accelerated both attachment and growth of cattle TE cells. Although the exact nature of factor(s) that fibroblast-CM may contain is not known, we have shown in an earlier study that transcripts of a number of growth factors and signalling molecules such *LIF*, *FGF-2*, *BMP-4*, *GREMLIN*, *NOGGIN*, *TGF-β1* and *ACTIVIN-A* were present in BFF feeder layer cells [30], as shown to be present in mouse and human feeder cells [31] also. Several of these factors may be present in fibroblast-CM and involved in stimulating the attachment and growth of TE cells.

Several characteristics of buffalo TE cells were found to be similar to those found in bovine TE cells [8,9]. Buffalo TE cells resembled bovine TE cells morphologically in terms of a flat, cuboidal appearance, granular cytoplasm and several lipid droplets, and like them these could not be completely dissociated by trypsinization due to which the colonies had to be dissociated into small pieces mechanically for subculture. Other common features included formation of dome-shaped structures following continuous culture under feeder-free conditions due to accumulation of fluid under the monolayer, and formation of distinct fluid-filled vesicles. The vesicles dissociated spontaneously from the colony and floated freely in the medium but became attached after being transferred to a new dish following which fresh outgrowths grew from the seeded vesicles. Buffalo TE cells had a centrally placed large prominent nucleus. The diameter and area of the TE cells and their nucleus-to-cytoplasm ratio was higher than that of fibroblast cells. In contrast, caprine trophoblast cell colonies derived from placental cells were reported to contain three different cell types [10]. Porcine TE cells also display a prominent nucleus and prominent lipid containing vesicles but do not form fluid-filled vesicles [11]. These differences could be due to species variation or difference in the origin of cells. Following immunofluorescence staining, buffalo TE cells were found to express both cytokeratin-18 and keratin but not vimentin, just like porcine TE cells [11]. Since bovine cells also express cytokeratin [9], TE cells across several species appear to be of epithelial and not fibroblast origin. TE cells were found to express markers reported to be specific for them [32] viz. *IFN-τ*, *CDX2*, *HAND1*, *GATA2*, *GATA3*, *ETS2*, *ELF-5*, *OCT4*, *PAG2* and *FGFR-2* by RT-PCR. In comparison, placental cells showed expression of *PAG2*, *CYTOKERATIN-8* and *-18*, *HAND1*, *GATA2*, *GATA3*, *ETS2* and *ELF-5* but not that of *CDX2* and *FGFR-2*. The expression of CDX2 was further confirmed to be present in blastocysts and TE cells by immunofluorescence staining. Despite wide variations in the regulatory circuitry determining ICM/TE identity, both CDX2 and GATA3, which are functionally related, are perhaps the best known markers for distinguishing TE from ICM cells across several species [33].

Very little information is available on the differences in expression levels of important genes among TE cells produced from different sources. GATA2 and GATA3 are transcription factors belonging to a family of structurally-related proteins which have been reported to be expressed in trophoblast cells in several species including mouse, human and bovine [34]. It has been shown that trophoblast-specific *ASCL2*, *CDX2*, *CSH1*, *ELF5* and *HAND1* genes were regulated by GATA2 and/or GATA3 in bovine cells and that endogenous *CDX2* and *IFN-τ* mRNAs were down-regulated by *GATA2* siRNA, while endogenous *ASCL2* and *CDX2* mRNAs were down-regulated by *GATA3* siRNA indicating that in addition to TE lineage specification, GATA2and/or GATA3 are involved in the regulation of trophoblast-specific gene transcription in bovine trophoblast cells [35]. However, we found that the expression level of *GATA2* and *GATA3* was not significantly different between TE-IVF and TE-HMC cells. Also, their expression level was found to be similar in TE-, AF- or FF-derived blastocysts or those produced by IVF.

Establishment and inheritance of tissue-specific methylation patterns require both d*e novo* and maintenance methylation which is carried out by DNA cytosine-5’-methyltransferase (DNMT) family of DNA methylases that includes DNMT1, 3a, and 3b. DNMT1 predominantly catalyzes maintenance methylation via binding to proliferating cell nuclear antigen in replication foci during S phase whereas DNMT3a and DNMT3b are mainly responsible for *de novo* methylation that establishes a new DNA methylation state at repeat sequences, imprinted genes, and developmental genes [36]. A systematic regulation of the activity of these enzymes is necessary for the establishment of appropriate methylation patterns. Occurrence of high levels of genomic DNA methylation is one of the important epigenetic abnormalities in cloned embryos produced by NT. We found the expression level of *DNMT1* to be lower in TE-HMC than in TE-IVF cells whereas that of *DNMT3a* was not significantly different. This pattern mimics that observed in 8-cell and blastocyst stage bovine embryos in which transcript levels of *DNMT1* were found to be significantly lower in NT embryos as compared to both IVF and parthenotes whereas the expression level of *DNMT3a* and *DNMT3b* was not different [37]. It was suggested that subsequent to embryonic genome activation, the transcriptional activity of DNMT3aand 3b is properly reprogrammed whereas Dnmt1 transcription is suppressed, likely as a response to the levels of hypermethylation in NT embryos and their potential impact upon methylation responsive control elements within the Dnmt1 promoter [37]. Our results indicate that this epigenetic pattern may be carried over to the TE cells derived from NT embryos. Extraembryonic tissues in general and placenta in particular are hypomethylated and have a globally lower level of genomic 5-methylcytosine than somatic tissues [38]. To what extent is the lower expression of *DNMT1* in NT embryos and TE cells responsible for this, requires further studies. Our results also showed that the epigenetic status of the donor cells, in terms of the global level of important markers H3K18ac and H3K27me3, was not reflected in the HMC embryos derived from them. This is due to the nuclear reprogramming of the donor cells which, besides altering DNA methylation patterns, results in changes in histones and other DNA accessory proteins which regulate the function of chromatin [39]. Different relative transcript levels of *DNMT3a*, which is responsible for *de novo* methylation, in blastocysts derived from TE, AF, FF and IVF blastocysts, may be a reflection of the different methylation status of embryos derived from different types of donor cells.

A lower expression level of *PAG1* but higher expression level of *PAG2* and *ETS2*, a gene involved in IFN-τ production by trophoblast cells [40] in TE-HMC than in TE-IVF cells, as seen in our study, may be of physiological significance since PAG proteins are believed to be luteoprotective chorionic-origin signals during implantation and placentation due to their ability to interact with gonadotropin receptors of luteal-phase animals [41]. A complex interplay between Ets2, Cdx2 and Hand1, a gene required for the differentiation of all trophoblast giant cells has been shown in recent studies [42]. Further studies are required on finding out the differences between trophoblast and placental cells of in vivo/IVF and cloned pregnancies to unravel the reasons behind abnormally high loss of cloned pregnancies. However, it is tempting to assume that the abnormal epigenetic profile of trophoblast cells derived from NT embryos, especially that of *DNMT1*, *HAND1*, *ELF5*, *PAG1*, *PAG2* and *ETS2* as observed in our study, is linked with the abnormal placenta associated with cloned pregnancies. We found that the pattern of expression level of many genes such as *DNMT1*, *DNMT3a*, *ETS2*, *PAG2* and *CDX2* in IVF and NT embryos reflected that present in the TE derived from them. It is, therefore, possible that the abnormal gene expression in the NT embryos is carried over to the TE cells and then to the placenta.

In the second part of the study, we used cells from the IVF-TE cell line as donor cells for producing NT embryos by HMC. In the only report available on the use of TE cells as donor cells [22], the blastocyst rate was found to be similar for bovine TE and AF cells (14.5 and 15.6%, respectively). However, we found the blastocyst rate of TE-derived embryos to be nearly 50% that of AF- and FF-derived embryos (20.7, 40.4 and 46.3%, respectively). Since we have consistently obtained a blastocyst rate of >40% from AF and FF in many of our previous studies [25,26,28]), the low blastocyst rate of TE-derived embryos points to their low developmental competence. Also, their quality was inferior as indicated by a higher apoptotic index than that of FF-derived HMC blastocysts or IVF blastocysts. We found that the expression level of *IFN-τ* was not significantly different among TE- and AF-derived and IVF blastocysts which agrees with the results of previous studies in which the expression levels of *IFN-τ* were found to be similar among bovine blastocysts produced by IVF, parthenogenesis or NT [20] or those produced in vivo or by IVF or NT [33]. However, in another study *IFN-τ* expression was found to be higher in TE-than in AF-derived NT or IVF blastocysts [22]. Others have reported that the in vitro produced embryos secreted more IFN-τ than those produced in vivo [43]. The differences in the results of various studies could be because expression and secretion of embryonic IFN-τ can be affected by many factors such as components of culture medium [44], procedures of embryo production [14] and sex of the embryo [15]. Even modifications of the NT protocol have been found to alter the expression pattern of *IFN-*τ in NT embryos compared to their IVP and in vivo-derived counterparts [45]. Nevertheless, these studies leave two important questions unanswered. First, does the expression level of IFN-τ in the Day 7 or Day 8 blastocysts reflect that at the time of implantation, which acts as a pregnancy recognition signal and second, to what extent do differences in IFN-τ production by pre-implantation embryos affect their ability to result in pregnancy following transfer to recipients. These issues warrant further investigations. We showed that the expression level of *IFN-*τ by TE cells was increased following supplementation of the culture medium with FGF2. Further studies need to be carried out to see if FGF2 is effective in increasing IFN-τ production by preimplantation embryos. Our results suggest that due to absence of high IFN-τ production combined with low developmental competence and quality of TE-derived NT embryos, TE cells do not offer any significant advantage over FF or AF as donor cells for NT.

Both OCT4 and CDX2 are involved in the regulation of IFN-τ production [46,47]. An increase in CDX2 negatively regulated OCT4 expression, but increased expression of IFN-τ, and a reduction of CDX2 levels exhibited a reciprocal effect whereas, in contrast to CDX2, manipulation of OCT4 levels only revealed a positive autoregulatory mechanism [48]. Our results, in terms of expression level of *OCT4* and *CDX2* and *in situ* expression of CDX2, which were higher in TE- than in FF-derived HMC blastocysts partly agree with those of Saadeldin et al. [22] who found the expression of CDX2 to be higher and that of OCT4 to be lower in TE- than that in AF-derived bovine blastocysts. Besides use of different methods for NT, this difference in results could be due to the use of semi-quantitative RT-PCR used by these authors in comparison to qPCR used by us.

Our results show that the epigenetic status of the donor cells, in terms of the global level of important markers H3K18ac and H3K27me3, was not reflected in the HMC embryos derived from them. This is due to the nuclear reprogramming of the donor cells which, besides altering DNA methylation patterns, results in changes in histones and other DNA accessory proteins which regulate the function of chromatin [39]. Different relative transcript levels of *DNMT3a*, which is responsible for *de novo* methylation, in blastocysts derived from TE, AF, FF and IVF blastocysts, may be a reflection of the different methylation status of embryos derived from different types of donor cells.

In conclusion, the results of the present study showed that although TE-HMC and TE-IVF cells have a similar capability to grow in culture, significant differences exist in gene expression levels between them and between IVF and NT embryos from which they are derived. This may have a role in the placental abnormalities associated with NT pregnancies. Although TE cells can be used as donor cells for producing NT blastocysts, their developmental competence and quality are lower and IFN-τ production is similar compared to that of blastocysts produced from fetal or adult fibroblasts. Therefore, TE cells do not offer any significant advantage over fetal or adult fibroblasts as donor cells for NT. Our results also showed that the epigenetic status and expression level of many important genes were different in NT blastocysts derived from TE cells or fetal or adult fibroblasts suggesting that these characteristics are dependent upon the nature of the donor cell.

## Supporting Information

S1 Table List of primers. (DOCX)

S1 Fig An IVF-derived blastocyst seeded on MaxGel-coated dish showing epiblast-derived fibroblasts. (A) Fibroblasts in primary TE colony at 40X; (B) at confluent stage at 200X; (C) showing positive expression for vimentin. Buffalo fetal fibroblasts characterized for vimentin expression by immunofluorescence staining (D-F). Scale bar = 100 μm.
(TIF)
