# Supplementary material for: Establishment of Trophectoderm Cell Lines from Buffalo (Bubalus bubalis) Embryos of Different Sources and Examination of In Vitro Developmental Competence, Quality, Epigenetic Status and Gene Expression in Cloned Embryos Derived from Them
Source: PLoS One. 2015 Jun 8;10(6):e0129235. doi: 10.1371/journal.pone.0129235 (PMC4459972; doi:10.1371/journal.pone.0129235)
Supplement: S1 Table — (DOCX) [file pone.0129235.s001.docx]

**S1 Table. List of primers.**

| **Sl no** | **Gene name** | **Accession no.** | **Size** | **AT** | **Primer sequences** |
| --- | --- | --- | --- | --- | --- |
| 1 | OCT-4 | EU926737 | 232 | 58 | F- GATATACCCAGGCCGATGTG  R-TCGATACTCGTCCGCTTTCT ’ |
| 2 | CDX-2 | NM_001206299.1 | 121 | 58 | F-GTCTGGAGCTGGAGAAGGAG  R- CTGCGGTTCTGAAACCAAAT |
| 3 | HAND1 | NM_001075761 | 191 | 58 | F-AGCACGCTAAGACGTAACCTTC  R-TCCCTGGCAGAAAACTATTGAT |
| 4 | IFN-tau | M60903 | 166 | 56 | F-CAGAAAAGACTTTGGTCTTCC  R-AGTGCAGAGCTGCTCCAGGAG |
| 5 | PAG1 | NM_174411.2 | 62 | 58 | F-TCCACTTTCCGGCTTACCAA  R-CCTTTCATTCTCCCAGATCCAT |
| 6 | FGFR2 | NM_001205310.1 | 280 | 58 | F-TACTTCATGGTCAATGTCACAG  R- GTTTCGTACCTTATAGCCT |
| 7 | GATA3 | NM_001076804.1 | 185 | 58 | F- ATGAAACCGAAACCCGATGG  R- TTCACAGCACTAGAGAGACC |
| 8 | CK 8 | NM_001033610.1 | 155 | 56 | F-CTTCAAGACCAAGTATGAGGA  R-TGCCTGTAGAAGTTGATCTC |
| 9 | CK 18 | NM_001192095.1 | 182 | 53 | F-CAGAGTCAAGTATGAGACAGAG  R-TGTAGACCCTTTACTTCCTCC |
| 10 | GATA2 | XM_583307.3 | 140 | 58 | F- GAGGACTGTAAGCGTAAAGG  R- AAGAACCAAGTCTCCCCAT |
| 11 | ELF5 | NM_001024569.1 | 147 | 56 | F-GCCTGTATCTCTGACTGTG  R-GGGTAATACTCTTCATTGCTG |
| 12 | ETS2 | NM_001080214.1 | 150 | 56 | F-CCGACCATGTCCTTCAAG  R-CTTGTCGGAGAGCAATTC |
| 13 | PAG2 | NM_176614.1 | 274 | 58 | F- GTGCCTTTTCTGAGCCTGTC  R- ATGAGCTTGTGGATGTTGG |
| 14 | DNMT3a | NM_001206502.1 | 188 | 58 | F-GTGCTGTCTCTATTCGATGG  R-CCATTCCTGGATATGCTTCTG |
| 15 | DNMT1 | NM_182651.2 | 138 | 58 | F-CTCAGAAGGGAGATGTGGAG  R-TAGTAGTCACAGTAGCTGAGGA |
| 16 | SOX2 | DQ487021 | 162 | 60 | F-TCCACATTCGAGATCAGCAA  R-CATGAGCGTCTTGGTTTTCC |
| 17 | GAPDH | GU32429.1 | 235 | 58 | F-ATCACCCTCGCTTCTACTGG  R-AGGAGACAACCTGGTCCTCA |

AT – Annealing Temperature
