# Supplementary figures and images for: Establishment of Trophectoderm Cell Lines from Buffalo (Bubalus bubalis) Embryos of Different Sources and Examination of In Vitro Developmental Competence, Quality, Epigenetic Status and Gene Expression in Cloned Embryos Derived from Them
Source: PLoS One. 2015 Jun 8;10(6):e0129235. doi: 10.1371/journal.pone.0129235 (PMC4459972; doi:10.1371/journal.pone.0129235)

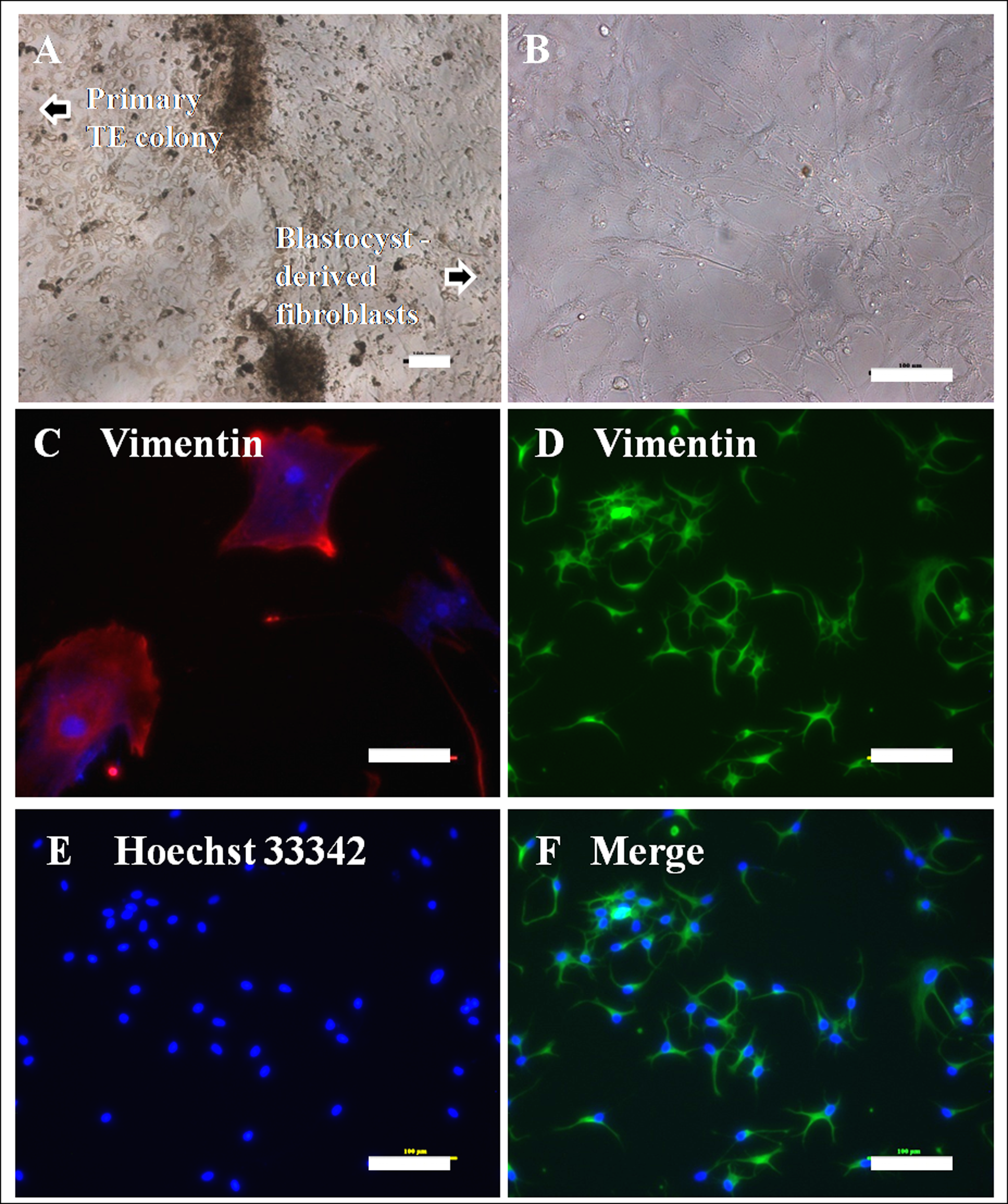

Supplement: S1 Fig — (A) Fibroblasts in primary TE colony at 40X; (B) at confluent stage at 200X; (C) showing positive expression for vimentin. Buffalo fetal fibroblasts characterized for vimentin expression by immunofluorescence staining (D-F). Scale bar = 100 μm. (TIF) [file pone.0129235.s002.tif]
